# Photoliquefaction and phase transition of *m*-bisazobenzenes give molecular solar thermal fuels with a high energy density†

**DOI:** 10.1039/d3ra04595a

**Published:** 2023-08-10

**Authors:** Masa-aki Morikawa, Yuta Yamanaka, Joseph Ka Ho Hui, Nobuo Kimizuka

**Affiliations:** a Department of Chemistry and Biochemistry, Graduate School of Engineering, Kyushu University 744 Moto-oka Nishi-ku Fukuoka 819-0395 Japan morikawa.masa-aki.973@m.kyushu-u.ac.jp kimizuka.nobuo.763@m.kyushu-u.ac.jp; b Center for Molecular Systems (CMS), Kyushu University 744 Motooka Nishi-ku Fukuoka 819-0395 Japan; c Research Center for Negative Emissions Technologies, Kyushu University (K-NETs) 744 Motooka Nishi-ku Fukuoka 819-0395 Japan

## Abstract

A series of *m*-bisazobenzene chromophores modified with various alkoxy substituents (1; methoxy, 2; ethoxy, 3; butoxy, 4; neopentyloxy) were developed for solvent-free molecular solar thermal fuels (STFs). Compounds (*E*,*E*)-1–3 in the crystalline thin film state exhibited photoliquefaction, the first example of photo-liquefiable *m*-bisazobenzenes. Meanwhile, (*E*,*E*)-4 did not show photoliquefaction due to the pronounced rigidity of the interdigitated molecular packing indicated by X-ray crystallography. The *m*-bisazobenzenes 1–4 exhibited twice the *Z*-to-*E* isomerization enthalpy compared to monoazobenzene derivatives, and the latent heat associated with the liquid–solid phase change further enhanced their heat storage capacity. To observe both exothermic *Z*-to-*E* isomerization and crystallization in a single heat-up process, the temperature increase of differential scanning calorimetry (DSC) must occur at a rate that does not deviate from thermodynamic equilibrium. Bisazobenzene 1 showed an unprecedented gravimetric heat storage capacity of 392 J g^−1^ that exceeds previous records for well-defined molecular STFs.

## Abstract 2

(*E*,*E*)-*m*-bisazobenzenes show photoliquefaction to liquid *Z*-bisazobenzenes, which reveal exothermicity due to *Z*-to-*E* isomerization and liquid-to-solid phase changes, with a high gravimetric heat storage capacity of up to 392 J g^−1^.

## Graphical abstract

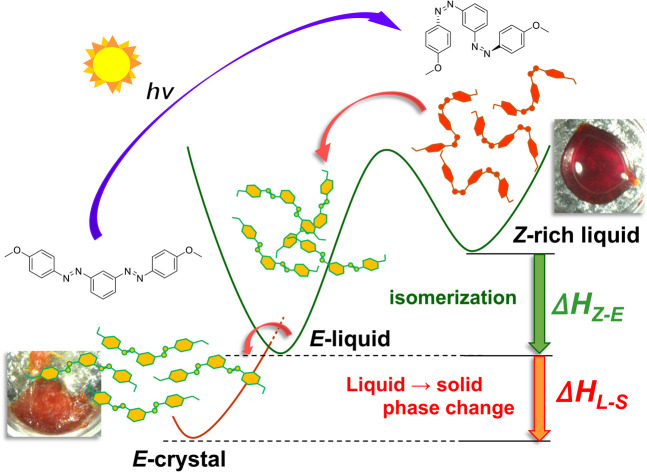

## 1. Introduction

Solar energy holds great promise as a sustainable and renewable energy source, and conversion and storage of solar energy into electric^1,2^ or chemical energy^3,4^ have been investigated worldwide. Meanwhile, there has been an urgent need for closed-cycle solar energy conversion technology, and molecular solar thermal fuels (STFs)^5–8^ have been attracting renewed attention.^9–13^ In molecular STFs, solar energy is stored in metastable photoisomers of photoisomerizable molecules as strain energies of chemical bonds, which are released as heat by applying external stimuli of light, heat or catalysis. It regenerates thermodynamically stable photoisomers without emitting environmentally harmful substances. Azobenzene derivatives have been intensively studied as photoresponsive STFs, due to their high thermal stability, vast molecular design possibilities, and ease of synthesis.^7,10,13–17^ Meanwhile, azobenzenes have fatal drawbacks. First, the isomerization enthalpy of *E*-azobenzenes is limited to *ca.* −50 kJ mol^−1^.^7,9^ Second, although the photoisomerization of azobenzene chromophores occurs in solution quickly, it has been significantly suppressed in the solid state.^9,18,19^ The dilution in solvents inevitably reduces the energy density and loses its value as molecular STFs.

To solve these problems, we made two innovations. First, a family of room-temperature liquid azobenzene and arylazopyrazole derivatives has been developed that show reversible photoisomerization in the solvent-free, condensed state.^20–22^ Liquid-azobenzene displayed photon energy storage with an isomerization enthalpy of −52 kJ mol^−1^,^20^ consistent with the pristine azobenzene chromophore dissolved in solution. Liquid arylazopyrazole showed a significantly long thermal half-life of the *Z*-isomer (*t*_1/2_ = 3069 h, at 25 °C).^21^ These liquid STFs do not need to be diluted in organic solvents, and the reduction in heat capacity based on dilution can be avoided. Second, to overcome the upper limit of the heat storage capacity that has traditionally been the molecular isomerization enthalpy, we introduced a liquid–solid phase transition in the molecular STF.^22^ This was achieved by developing photoliquefiable azobenzene crystals (A1, Scheme S1†) that successfully add latent heat to the isomerization enthalpy, producing an overall heat storage capacity of −97 kJ mol^−1^. This study created new molecular design guidelines for photoresponsive phase-change STFs that exceeded the limits of molecular isomerization enthalpy, setting the direction for subsequent research in this field.^13,23,24^

Meanwhile, in many reports by other researchers, the isomerization enthalpy of thermally induced *Z*-to-*E* isomerization has been observed during the differential scanning calorimetry (DSC) heating process, and the latent heat associated with the phase change (*i.e.*, the heat of solidification) has been observed separately during the cooling process.^23,26,32^ This is because the heating rates employed (∼10 °C min^−1^) are faster than the crystallization rate, and the energy storage densities reported for these compounds are only the mathematical sum of the exothermic peaks obtained during heating and cooling.^23,26,32^ For the practical application of phase transition STFs, however, it is desirable to achieve both exothermic phenomena simultaneously in a single heat-up scan.^22^ It simplifies the process and minimizes the electrical energy required to obtain exothermic energy. In this regard, we point out that DSC should be performed at an appropriate heating rate that does not deviate from thermodynamic equilibrium.

In addition to these issues, the next challenge is to increase the gravimetric energy density of the condensed phase STF, where performance above 300 J g^−1^ is desired for practical use.^25^ This energy density can be met using small molecular weight molecules that exhibit photoinduced phase transitions, but examples are extremely limited,^26,27^ and no rational design guidelines have been obtained. For this, it is reasonable to introduce multiple photoswitching units into a molecule.^28,29^ Moth-Poulsen *et al.* have reported an improved energy density for dimeric and trimeric norbornadiene/quadricyclane (NBD/QC) pairs in solution.^28^ An increase in the energy density of STFs was theoretically predicted for bisazobenzene derivatives immobilized on templates,^29^ but the isomerization enthalpy determined for a powdery *Z*-1,3,5-tris(arylazo)benzene remained low (111–139 J g^−1^, −43 to −54 kJ mol^−1^).^30^ Wegner *et al.* reported azobenzene derivatives connected *via* molecular linkers, which showed isomerization enthalpy per azobenzene unit (−44 to −55 kJ mol^−1^) almost similar to that of pristine azobenzene (−48 kJ mol^−1^).^31^ Wu *et al.* recently reported the heat storage capacity of *Z*-tris(azobenzene) compounds with methoxy substituents to be 242 J g^−1^ (=−115 kJ mol^−1^, ∼−38 kJ per azobenzene unit), which is still not significantly different from that of 4-methoxy azobenzene (255 J g^−1^, −54 kJ mol^−1^).^32^ This is ascribed to the low *E*-to-*Z* photoisomerization efficiency of the tri-azobenzene compound, the instability of the *Z* form, and the absence of photoliquefaction characteristics.

**Scheme 1:**
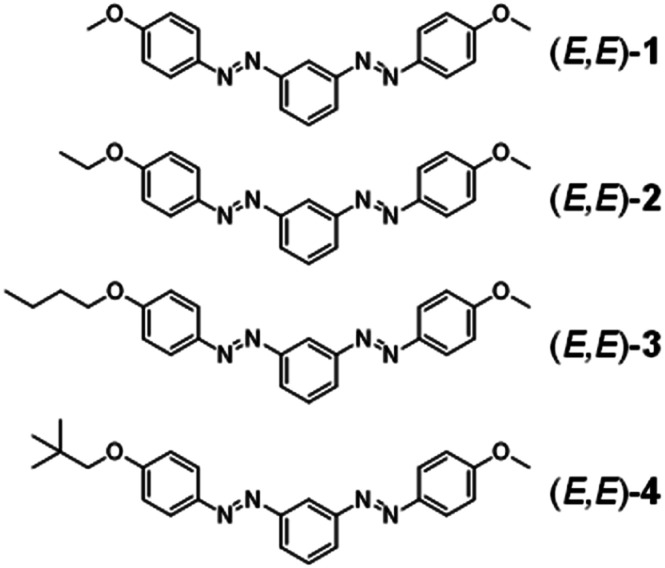
Chemical structure of *m*-bisazobenzene derivatives (*E*,*E*)-1–4.

To develop condensed-phase STFs with a gravimetric energy density as high as possible, we have recently reported liquid *m*-bisazobenzenes,^33^ in which two azo groups share one phenyl group in the meta position (A2, Scheme S1†). It showed a heat storage capacity of 230 J g^−1^, more significant than the mono-azobenzene liquid STF (168 J g^−1^).^20^ The introduction of a solid–liquid phase transition in bisazobenzene derivatives is expected to improve their STFs properties further. In this work, we report the development of photoliquefiable bisazobenzene compounds that show a photoinduced crystal-to-liquid phase transition (Scheme 1).

According to the previous work, we adopted the *m*-bisazobenzene unit because each azobenzene chromophores show independent photoisomerization properties. In compound 1, methoxy groups are introduced symmetrically at both ends of the molecule to enhance the volume and weight densities by keeping the molecular weight as small as possible. In compounds 2–4, alkoxy substituents of different alkyl chain lengths were introduced asymmetrically into the banana-shaped *m*-bisazobenzene structure to tune the molecular packing and cohesive forces that influence photoliquefaction. By introducing the latent heat associated with the crystallization of photoliquefied *Z*-isomers, we successfully developed bis-azobenzene STFs with an unprecedentedly high energy density.

These features are not accessible from the *p*-bisazobenzene compounds.^34–36^ Until now, the relationship between the photoliquefaction phenomena and molecular orientation in crystals has not been fully understood. In this study, good single crystals were obtained for (*E*,*E*)-1 and (*E*,*E*)-4, and analysis of their single-crystal X-ray structure revealed a correlation between photoisomerization properties and crystal structure.

## 2. Materials and methods

### 2.1 Materials

DMF was freshly distilled before use. All chemicals from commercial sources were used as received unless otherwise stated. Phenol, 3′-aminoacetanilide, and 1-bromobutane were purchased from TCI, 1-bromo-2,2-dimethylpropane, magnesium sulfate, sodium nitrite, potassium iodide, iodoethane, and iodomethane were purchased from FUJIFILM Wako Pure Chemical Corporation, 37% hydrochloric acid, sodium hydroxide, and potassium carbonate were purchased from KISHIDA Chemical Co., Ltd.

### 2.2 General methods

^1^H NMR (400 MHz) spectra were recorded on a JEOL JNM-ECZ400 spectrometer using TMS as the internal standard. Elemental analysis was conducted using a Yanaco CHN Corder MT-5 at the Elemental Analysis Center, Kyushu University. UV-vis absorption spectra were recorded on a JASCO V-770 UV-vis-NIR spectrophotometer. Differential scanning calorimetry (DSC) experiments were performed on a Mettler Toledo DSC-1 under a nitrogen atmosphere. Photoillumination was performed using a xenon lamp (MAX-350, Asahi Spectra) and a super high-pressure mercury lamp (USH-500D, USHIO) with bandpass filters (FWHM: 10 nm). A xenon lamp was used for photoisomerization experiments of sample solutions. In the light irradiation experiments, the temperature of the samples was kept at 20 °C using a temperature controller. The power density of the illumination light as a function of the wavelength is shown in Fig. S30.† Photoisomerization from *E*-to-*Z* and from *Z*-to-*E* in microspectrophotometry (in Fig. 2 and S18–S20†) was performed with a Nikon C-SHG1 super-high-pressure mercury lamp (power density > 6.2 mW cm^−2^). An USHIO USH-500D super high-pressure mercury lamp (power density ∼ 300 mW cm^−2^) was used for the other UV irradiation experiments (XRD and DSC samples, Fig. 3 and S25–S29†). Photoisomerized *Z* compounds were used almost immediately and stored in the dark to prevent *Z*-to-*E* photoisomerization.

The *Z*-to-*E* reversion of compounds 1–4 in acetonitrile solution was studied in the dark at 20, 30, 40, 50, and 60 °C. The thermal relaxation rate was monitored at the maximum absorption of the *E*-isomer, and the process was found to be of the first order, as shown by the linear plots in Fig. S14a–S17a.† Activation parameters, such as enthalpy (Δ*H*^‡^), entropy (Δ*S*^‡^), and free energy of activation (Δ*G*^‡^), were determined by measuring the temperature dependence of the rate constant and fitting the data with the Eyring equation (Fig. S14b–S17b†).

## 3. Results and discussion

### 3.1 Photoisomerization in solution

*m*-Bisazobenzenes (*E*,*E*)-1–4 were obtained as crystalline solids at room temperature. Differential scanning calorimetry (DSC) measurements (Fig. S1 and Table S1†) showed that the melting point decreases with increasing the carbon number of *n*-alkoxy substituents ((*E*,*E*)-1; 110.5 °C, (*E*,*E*)-2; 109.1 °C, (*E*,*E*)-3; 86.9 °C). The decrease in melting point with an increasing carbon number of alkoxy groups can be understood by the increase in entropy in the liquid state due to the increased rotational and vibrational degrees of freedom of the alkyl chains. Meanwhile, compound (*E*,*E*)-4 with the neopentyloxy group exhibited the highest melting point at 119.8 °C. As discussed later, this will be attributable to the interdigitated antiparallel molecular packing in the crystal, which is distinct from those of the other compounds ((*E*,*E*)-1–3).

**Fig. 1:**
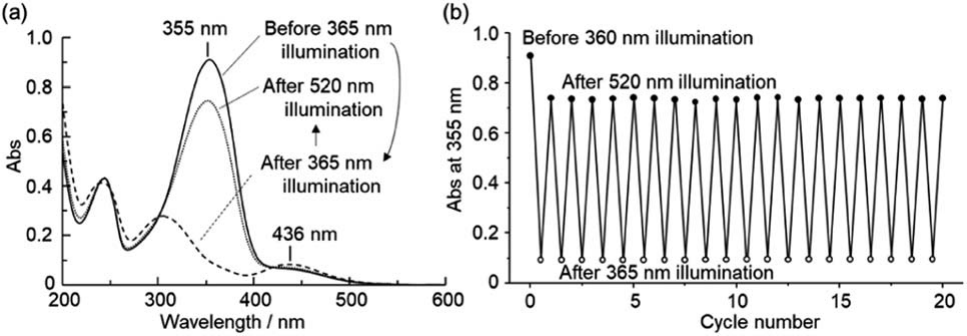
Photoisomerization properties of bisazobenzene derivatives (1) in acetonitrile solution (concentration; 20 μM, 25 °C). (a) UV-vis absorption spectra of (*E*,*E*)-1 before (solid line) and after photoillumination at 365 nm (dashed line) and 520 nm (dotted line) for 3 min. (b) Changes in absorbance at 355 nm during repetitive photoillumination at 365 nm (open circle) and 520 nm (filled circle) for 3 min.

First, the basic photoisomerization characteristics of each compound were investigated in dilute acetonitrile solutions. Fig. 1a shows the UV-vis absorption spectra of *m*-bisazobenzene 1. Before photoirradiation, (*E*,*E*)-1 showed a band with a maximum at 355 nm (solid line), which is assigned to the π–π* transition (S_0_–S_2_) of the *E*-azobenzene chromophore. After irradiation of the solution with 365 nm light, the absorption intensity at 355 nm decreased and changed to absorption with maxima at 305 and 436 nm (broken line), the latter assigned to the n–π* transition (S_0_–S_1_) of the *Z*-azobenzene unit. Meanwhile, when irradiated with 520 nm light, the spectrum returned to the original spectral pattern with isosbestic points at 300 and 419 nm, respectively. Such spectral changes are typical of the *E*-to-*Z* and *Z*-to-*E* photoisomerization of azobenzene. The excitation wavelengths for the *E*-to-*Z* and *Z*-to-*E* photoisomerization were chosen with reference to the UV-vis spectra obtained after reaching the photostationary state (PSS) at various excitation wavelengths (Fig. S2†).

As *m*-bisazobenzene (*E*,*E*)-1 is converted to a mixture of the three possible geometric isomers (*E*,*E*), (*E*,*Z*) and (*Z*,*Z*), the ratio of each isomer was determined by ^1^H-NMR spectroscopy (Fig. S3†). The fraction of each isomer that exists in the PSS at various excitation wavelengths is shown in Fig. S4.† Maximal conversion to the *Z* isomer (93%) and the *E* isomer (74%) were found to be achieved at excitation wavelengths of 365 and 520 nm, respectively (Table S2†). The photoisomerization of *E*-to-*Z* and *Z*-to-*E* proceeds reversibly, and the absorbance at each PSS remains unchanged after repeated light irradiation (Fig. 1b). Similarly, reversible photoisomerization properties were also observed for other derivatives of *m*-bisazobenzene (2–4) (Fig. S5–S13†), underlining their photochemical stability. The half-lives of metastable *Z* isomers and their thermodynamic activation parameters in acetonitrile were investigated as described in ESI (Fig. S14–S17 and Table S3†).

### 3.2 Photoisomerization in the solid state

**Fig. 2:**
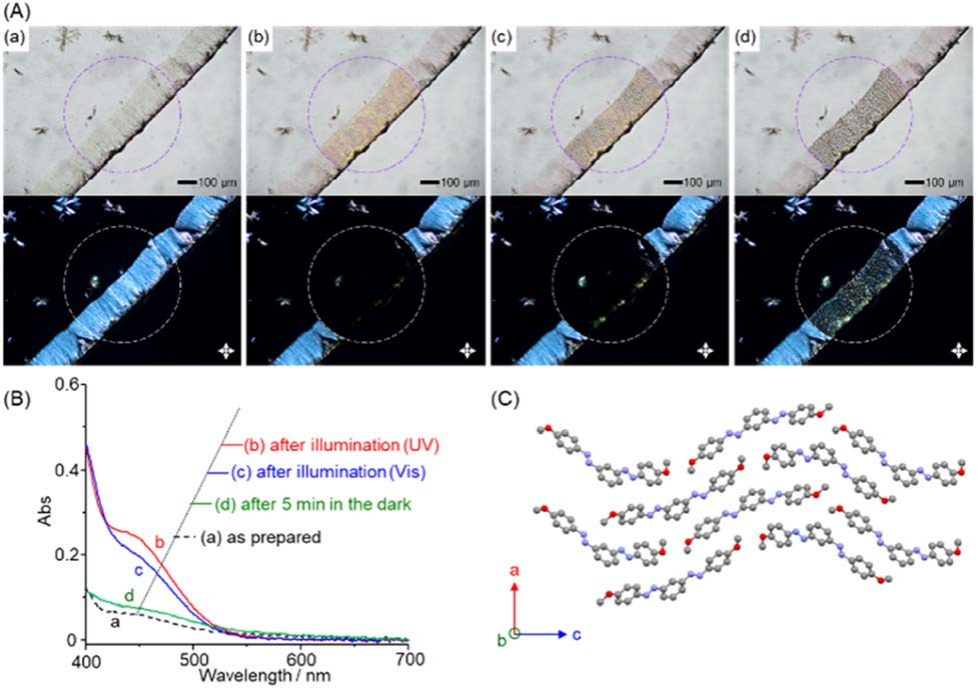
(A) Optical microscopy images (top; bright field, bottom; under crossed polarizers) and (B) visible absorption spectra of drop cast film of 1 on a glass substrate. (a) As prepared, (b) after photoillumination with a mercury lamp (bandpass filter: 380–420 nm) for 5 s, (c) after photoillumination (bandpass filter: 510–560 nm) for 5 s, and (d) after standing for 5 min in the dark. (C) The crystal structure of (*E*,*E*)-1 along the *b*-axis.

We then examined the photoisomerization properties of *m*-bisazobenzene derivatives in the solid state. A methanol solution of (*E*,*E*)-1 (1 mM) was cast onto a glass slide and dried under a vacuum. The thin solid film formed near the edge of the glass slides was observed by the bright field (top) and polarized optical microscopy (POM) under crossed polarizers (bottom, Fig. 2A). Before photoirradiation (a), the POM image shows birefringence, indicating that the sample is a crystalline film. On the other hand, when a high-pressure mercury lamp was irradiated through a 380–420 nm bandpass filter for 5 s (b), the birefringence in the photoirradiated area (circle in the figure) disappeared, and the crystalline thin film liquefied into an isotropic liquid. Changes in visible absorption spectra associated with this photoliquefaction process were obtained by microspectroscopy (Fig. 2B), which showed an increase in the absorption intensity of the S_0_–S_1_ absorption around 450 nm (Fig. 2B, a→b), indicating that photoliquefaction occurred as a result of *E*-to-*Z* photoisomerization in the crystalline state. Meanwhile, when the same region was irradiated through a bandpass filter at 510–560 nm for 5 s, the absorption intensity around 450 nm decreased (b→c), indicating the occurrence of the reverse *Z*-to-*E* photoisomerization. Microscopic observations confirmed that crystallization began, but most of the product remained in the liquid phase. On the other hand, after 5 minutes in the dark, the crystallization progressed, and the spectrum returned to that of the pristine crystal (*E*,*E*)-1 (Fig. 2B, c→d). The observed time lag is assumed to be due to the influence of the glass slide surface on the thin crystals. These results indicate that bisazobenzene (*E*,*E*)-1 in its crystalline form exhibits a photoinduced phase change to the liquid phase, including the Z-forms, upon photoisomerization. When compiling this study into a paper, we found the photoisomerization of compound (*E*,*E*)-1 in solution reported by Wu *et al.*^32^ They described that the powder sample of (*E*,*E*)-1 did not show photoisomerization; instead, only a change in colour on the powder surface was observed. As described above, the *E*-to-*Z* photoisomerization of azobenzenes is generally suppressed in crystals because it increases the molecular volume. Even for azobenzene derivatives showing photoisomerization in the crystalline state, the irradiated light is preferentially absorbed by chromophores near the surface. The light penetration depth of 365 nm light is only *ca.* 1–2 μm,^27^ and the efficiency and rate of photoisomerization depend on the thickness of the samples and the ability of the generated liquid *Z*-form to solubilize the *E*-form in the crystal domain and promote its *E*-to-*Z* photoisomerization by convection. Therefore, we presume that the thick powder samples^32^ limited light penetration, which was not suitable for identifying the photoliquefaction ability. By employing thin solid films, we also confirmed the generality of the photoliquefaction phenomenon for the other *m*-bisazobenzene derivatives.

In the case of compound (*E*,*E*)-2 with an ethoxy group, photoirradiation of the crystals for 5 s caused an apparent colour change with partial photoliquefaction (Fig. S18†). Even with a longer light irradiation period (10 min), the entire sample was not liquefied and the formation of microcrystals was observed from the liquid domain. We assume that the *E*-form present in the photostationary state phase separates to give microcrystals, and it is related to the limited miscibility of *E*- and *Z*-forms of 2. Meanwhile, the liquid phase of the *Z*-form facilely crystallized upon illumination of the 510–560 nm light. Compound (*E*,*E*)-3, which has a butoxy group, showed rapid photoliquefaction within 5 s. Still, the supercooled *E*-liquid phase was relatively stable, and the crystallization occurred partially after standing for 60 min in the dark (Fig. S19†). In contrast, compound (*E*,*E*)-4, having a neopentyloxy group, did not show changes in the microscopic image or the absorption spectrum upon photoirradiation (Fig. S20†), indicating that compound (*E*,*E*)-4 is not photoresponsive in the solid state. Thus, the photoisomerization and phase change properties in the crystalline system highly depend on the structure of substituents introduced to the *m*-bisazobenzene chromophore.

Single-crystal XRD was conducted for compounds (*E*,*E*)-1–4 to gain further insight into the observed differences in the photoisomerization properties. All the compounds gave monoclinic crystalline structures with similar molecular densities as determined by the lattice parameters. ((*E*,*E*)-1, 1.33 g cm^−3^, (*E*,*E*)-2, 1.31 g cm^−3^, (*E*,*E*)-3, 1.29 g cm^−3^, (*E*,*E*)-4, 1.27 g cm^−3^). Good single crystals and analysis data were obtained for (*E*,*E*)-1 and (*E*,*E*)-4. Compounds (*E*,*E*)-2 and (*E*,*E*)-3, meanwhile, did not provide fine single crystals suitable enough for structural analysis. We, however, show the data for (*E*,*E*)-2 and (*E*,*E*)-3 as references. Compound (*E*,*E*)-1, which underwent photoliquefaction, gave a crystal structure with banana-shaped molecules arranged in the *P*2_1_ space group (Fig. 2C and S21†). The two azobenzene groups in molecule (*E*,*E*)-1 are in edge-on orientation rather than π–π stacking with the azobenzene groups of different neighbouring molecules. Compound (*E*,*E*)-2, which showed partial liquefaction, revealed two pairs of slip-stacked molecules in the unit cell (Fig. S22†). The facilely photoliquefiable (*E*,*E*)-3 shows dimer units arranged in the unit cell (Fig. S23†). On the other hand, (*E*,*E*)-4, which does not show photoisomerization or liquefaction behaviour, showed an interdigitated antiparallel molecular orientation that can mitigate steric repulsion between bulky neopentyl groups (Fig. S24†). Thus, these *m*-bisazobenzene compounds (*E*,*E*)-1–4 take various molecular orientations in crystals depending on the substituents. The crystal densities of (*E*,*E*)-1–3 exhibit photoliquefaction in the crystalline state, and those of (*E*,*E*)-4, which do not photoisomerize, do not differ significantly. The *E*-to-*Z* photoisomerization requires an increase in the molecular volume, which is governed by the sum of intermolecular interactions determined by the crystal's microscopic molecular orientation and packing. The interdigitated molecular orientation observed in (*E*,*E*)-4 exhibits high intermolecular forces that suppress photoisomerization, as indicated by its higher melting point than the other compounds.

### 3.3 Photon energy storage properties

**Fig. 3:**
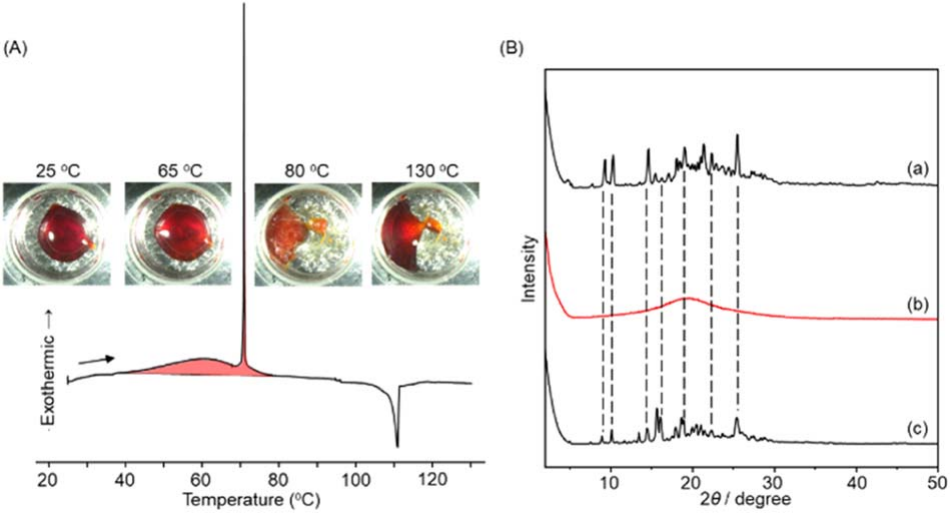
(A) DSC thermogram of 1 after UV light irradiation. The total percentage of *Z*% (86%) was determined by ^1^H-NMR spectroscopy. Heating rate, 0.2 °C min^−1^. The inset shows photographs of the DSC samples at each temperature. (B) Powder X-ray diffraction patterns obtained for 1 prepared on a glass substrate: (a) hot melted at 150 °C and allowed to stand at room temperature (total *E*%: 100%), (b) after UV irradiation at room temperature (total *Z*%: 83%), and (c) after standing at 60 °C overnight (total *E*%: 100%). A high-pressure mercury lamp was used for UV illumination (*λ* = 365 ± 10 nm, power density; ∼300 mW cm^−2^).

The photon energy storage properties of the *m*-bisazobenzene derivatives were then investigated by determining the enthalpy released during the thermally induced *Z*-to-*E* isomerization. (*E*,*E*)-1 was dissolved in dichloromethane and photoisomerized to the *Z* isomer by irradiating at 365 nm using a high-pressure mercury lamp (power density; ∼300 mW cm^−2^). The *Z* isomer content of 86% was determined by ^1^H-NMR spectroscopy for the viscous liquid obtained after vacuum drying, which showed no diffraction peaks in the PXRD measurement (Fig. 3B(b)). No crystallization occurred even after cooling the photoisomerized *Z*-1 liquid from 30 °C to −100 °C, and only a glass transition was observed around −70 °C on the DSC thermogram (Fig. S25†).

These observations indicate that *Z*-1 is essentially in a liquid state. Meanwhile, when the photoliquefied *Z*-1 was heated, the DSC curve for the first heating trace showed the coexistence of two exothermic peaks; a broad exothermic peak starting from 40 °C and a peak positioned around 60 °C and a successive sharp exothermic peak at 71 °C (Fig. 3A). An endothermic peak observed at 110 °C is attributed to the melting point of (*E*,*E*)-1 (Fig. S1a†). The inset in Fig. 3A shows the photographs of the DSC samples at each temperature during heating. The sample remained liquid at 65 °C and crystallized at 80 °C. Further heating to 130 °C resulted in the melting of the sample. The occurrence of thermal *Z*-to-*E* isomerization was confirmed for the *Z*-rich liquid kept at 60 °C overnight, which showed diffraction peaks in PXRD consistent with the original crystals (*E*,*E*)-1 (Fig. 3B(a) and (c)). These results indicate that the broad exothermic peak is associated with the enthalpy release due to thermal *Z*-to-*E* isomerization, and the sharp exothermic peak originates from the latent heat associated with the crystallization of (*E*,*E*)-1. This behaviour is similar to those observed for photoliquefiable azobenzene ionic crystals.^22^ The combined heat storage capacity for *Z*-rich 1 was determined as 116.8 kJ mol^−1^ (337 J g^−1^), which corresponds to 135.8 kJ mol^−1^ (392 J g^−1^) for 100% *Z*-isomer. These values are much higher than the previously reported gravimetric heat storage capacity of liquid *m*-bisazobenzene (230 J g^−1^).^33^

It should be noted that DSC is used to obtain thermodynamic parameters related to thermal equilibrium. Therefore, it is crucial to properly set the heating rate to determine the heat storage and generation of heat using DSC.^22^ Fig. S26–S29† compare the DSC thermograms obtained by heating photoisomerized *Z*-rich forms of 1–4 at a heating rate of 1 °C min^−1^ (a) and 0.2 °C min^−1^ (b). At a slow heating rate of 0.2 °C min^−1^, all samples showed both the broad exotherms (40–80 °C) based on thermal isomerization from *Z*-to-*E* and sharp exothermic peaks originating from the crystallization of the *E*-isomers around 70 °C. On the other hand, at a higher heating rate of 1 °C min^−1^, only broad exothermic peaks (50–90 °C) due to the thermal *Z*-to-*E* isomerization were observed without the sharp crystallization peaks of the *E*-isomer. The thermally induced crystallization process of (*E*,*E*)-1 would involve the formation of crystal nuclei and subsequent crystal growth processes. These rates are lowered in the dense, neat liquid phase, and the crystallization rate cannot keep up with the temperature and thermal motion increase at a high heating rate. Thus, for phase-transition-based molecular STFs, the heating rate is an essential parameter, and the time-axis parameters that include the crystallization rate need to be considered.^22^

**Fig. 4:**
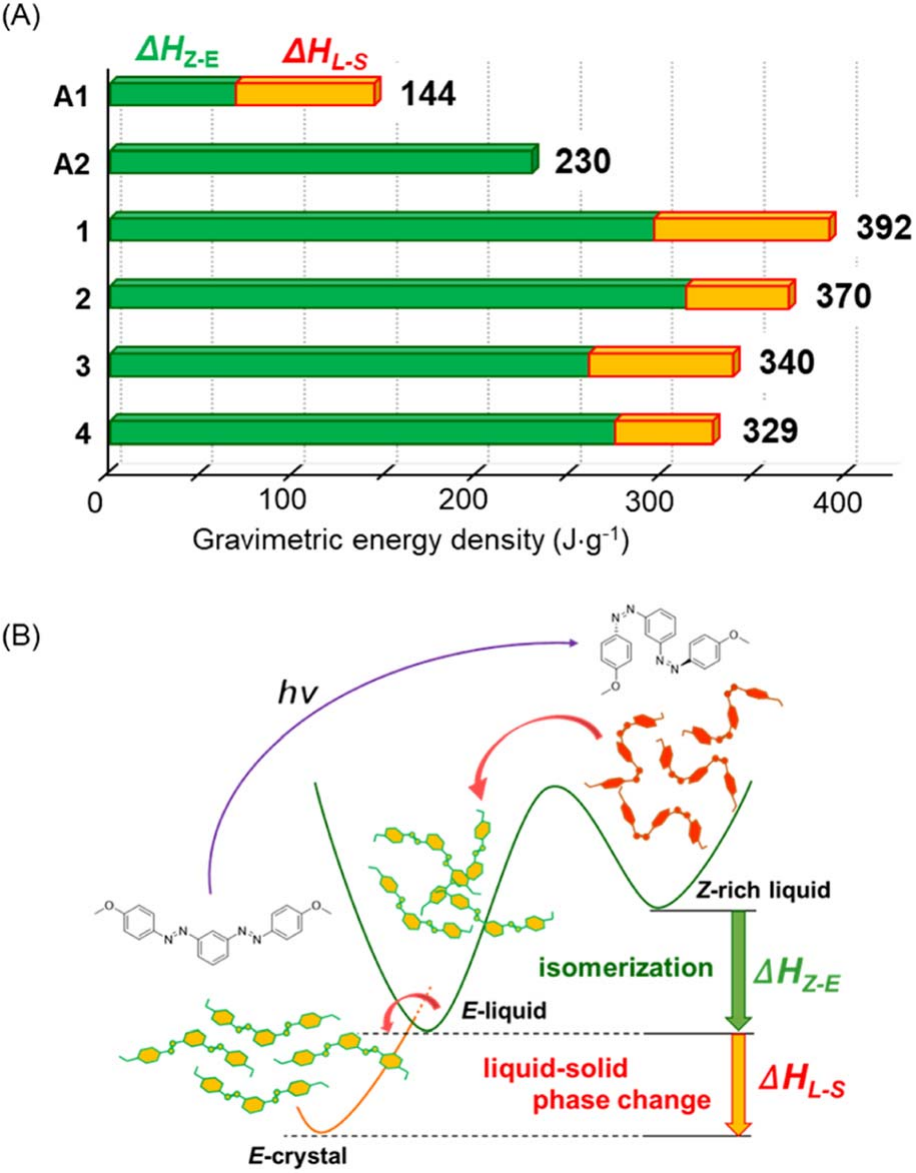
(A) The total gravimetric energy density is shown as a sum of the isomerization enthalpy and the *E*-isomer crystallization enthalpy estimated from the exothermic peaks in the first heating process of DSC measurements. Compounds 1–4 and two reported compounds A1 and A2 are shown for comparison. (B) Schematic illustration of the thermally induced *Z*-to-*E* isomerization of *m*-bisazobenzenes and their crystallization processes. Both the *Z*-to-*E* isomerization enthalpy (Δ*H*_*Z*–*E*_) and the latent heat of phase change (Δ*H*_L–S_) are continuously obtained in a single heating step.

Finally, we compared the gravimetric energy densities of the *m*-bisazobenzene STFs (Fig. 4 and Table S4†). The results of the previously reported ionic azobenzene derivative (A1, Scheme S1†)^22^ and liquid bisazobenzene with the 2-ethylhexyloxy group (A2, Scheme S1†)^33^ are also compared. Compared to the monoazobenzene derivative, A1, the isomerization enthalpy (green bar) for the bisazobenzene derivatives (A2 and 1–4) was significantly enhanced. Furthermore, the heat storage capacities of compounds 1–4 were further increased by the additional latent heat of the phase changes (orange bar), which is obtained in a single heating operation. Compound 1, with the smallest molecular weight, had the most significant gravimetric energy density of 392 J g^−1^. To our knowledge, this value is the record for the gravimetric heat storage capacity in pure and well-defined azobenzene-related molecular STFs.^23,26,27^

## 4. Conclusions

In conclusion, this study has unraveled the relationship between the molecular structure and molecular packing of *m*-bisazobenzene derivatives, their photoliquefaction and molecular STF properties. Photoisomerization from *E* to *Z* occurred almost quantitatively upon UV irradiation in solution, and the *Z* isomer showed sufficient thermal stability. Thin solid films of compounds (*E*,*E*)-1, (*E*,*E*)-2, and (*E*,*E*)-3 showed photoliquefaction properties, with compounds 1 and 3 being the most easily photoliquefiable. Meanwhile, compound (*E*,*E*)-4, which has the bulkiest neopentyloxy group, did not show photoliquefaction, and the suppressed photoisomerization in the solid state is ascribable to the rigid interdigitated molecular packing. Thermally induced *Z*-to-*E* isomerization and crystallization of (*E*,*E*)-rich isomers proceeded relatively easily for 1 but slowly for 3, indicating that the chemical structure of substituents has an essential influence on the photoliquefaction and thermally induced kinetic crystallization phenomena. To simultaneously observe the *Z*-to-*E* isomerization heat and the latent heat of crystallization in a single DSC heat-up process, the heating rate must be slow enough to maintain thermodynamic equilibrium. The heat storage capacity of bisazobenzene derivatives was improved over that of the monoazobenzenes, and the introduction of the solid–liquid phase transition further enhanced the heat storage capacity. An unprecedented gravimetric storage heat capacity of 392 J g^−1^ was observed for 1, which exceeds the previous records for well-defined molecular STFs. The results of this study provide a valuable molecular design principle for next-generation high-energy-density solar thermal batteries.

## Author contributions

M. M. and N. K. designed the project. Y. Y. and M. M. conducted the experimental work. J. K-H. H. conducted the single-crystal XRD measurement and analysis. M. M., J. K-H. H. and N. K. co-wrote the paper.

## Conflicts of interest

There are no conflicts to declare.

## Supplementary Material

RA-013-D3RA04595A-s001

RA-013-D3RA04595A-s002

## References

[cit1] Hagfeldt A., Grätzel M. (2000). Acc. Chem. Res..

[cit2] Kojima A., Teshima K., Shirai Y., Miyasaka T. (2009). J. Am. Chem. Soc..

[cit3] Lewis N. S., Nocera D. G. (2006). Proc. Natl. Acad. Sci. U. S. A..

[cit4] Takata T., Jiang J., Sakata Y., Nakabayashi M., Shibata N., Nandal V., Seki K., Hisatomi T., Domen K. (2020). Nature.

[cit5] Jones G., Chiang II S.-H., Xuan P. T. (1979). J. Photochem..

[cit6] Scharf H.-D., Fleischhauer J., Leismann H., Ressler I., Schleker W., Weitz R. (1979). Angew. Chem., Int. Ed..

[cit7] Taoda H., Hayakawa K., Kawase K., Yamakita H. (1987). J. Chem. Eng. Jpn..

[cit8] Yoshida Z. (1985). J. Photochem..

[cit9] Kucharski T. J., Tian Y., Akbulatov S., Boulatov R. (2011). Energy Environ. Sci..

[cit10] Kimizuka N., Yanai N., Morikawa M.-a. (2016). Langmuir.

[cit11] Sun C.-L., Wang C., Boulatov R. (2019). ChemPhotoChem.

[cit12] Orrego-Hernández J., Dreos A., Moth-Poulsen K. (2020). Acc. Chem. Res..

[cit13] Qiu Q., Shi Y., Han G. G. D. (2021). J. Mater. Chem. C.

[cit14] Wang Z., Moïse H., Cacciarini M., Nielsen M. B., Morikawa M.-a., Kimizuka N., Moth-Poulsen K. (2021). Adv. Sci..

[cit15] Dong L., Feng Y., Wang L., Feng W. (2018). Chem. Soc. Rev..

[cit16] Kolpak A. M., Grossman J. C. (2011). Nano Lett..

[cit17] Kucharski T. J., Ferralis N., Kolpak A. M., Zheng J. O., Nocera D. G., Grossman J. C. (2014). Nat. Chem..

[cit18] Ichimura K. (2009). Chem. Commun..

[cit19] Koshima H., Ojima N., Uchimoto H. (2009). J. Am. Chem. Soc..

[cit20] Masutani K., Morikawa M.-a., Kimizuka N. (2014). Chem. Commun..

[cit21] Morikawa M.-a., Yang H., Ishiba K., Masutani K., Hui J. K.-H., Kimizuka N. (2020). Chem. Lett..

[cit22] Ishiba K., Morikawa M.-a., Chikara C., Yamada T., Iwase K., Kawakita M., Kimizuka N. (2015). Angew. Chem., Int. Ed..

[cit23] Zhang Z.-Y., He Y., Wang Z., Xu J., Xie M., Tao P., Ji D., Moth-Poulsen K., Li T. (2020). J. Am. Chem. Soc..

[cit24] Le M., Han G. G. D. (2022). Acc. Mater. Res..

[cit25] Dreos A., Börjesson K., Wang Z., Roffey A., Norwood Z., Kushnir D., Moth-Poulsen K. (2017). Energy Environ. Sci..

[cit26] Qiu Q., Gerkman M. A., Shi Y., Han G. G. D. (2021). Chem. Commun..

[cit27] Gonzalez A., Odaybat M., Le M., Greenfield J. L., White A. J. P., Li X., Fuchter M. J., Han G. G. D. (2022). J. Am. Chem. Soc..

[cit28] Mansø M., Petersen A. U., Wang Z., Erhart P., Nielsen M. B., Moth-Poulsen K. (2018). Nat. Commun..

[cit29] Kolpak A. M., Grossman J. C. (2013). J. Chem. Phys..

[cit30] Dong L., Chen Y., Zhai F., Tang L., Gao W., Tang J., Feng Y., Feng W. (2020). J. Mater. Chem. A.

[cit31] Kunz A., Heindl A. H., Dreos A., Wang Z., Moth-Poulsen K., Becker J., Wegner H. A. (2019). ChemPlusChem.

[cit32] Sun S., Liang S., Xy W.-C., Wang M., Gao J., Zhang Q., Wu S. (2022). Soft Matter.

[cit33] Morikawa M.-a., Yamanaka Y., Kimizuka N. (2022). Chem. Lett..

[cit34] Cisnetti F., Ballardini R., Credi A., Gandolfi M. T., Masiero S., Negri F., Pieraccini S., Spada G. P. (2004). Chem.–Eur. J..

[cit35] Vapaavuori J., Goulet-Hanssens A., Heikkinen I. T. S., Barrett C. J., Priimagi A. (2014). Chem. Mater..

[cit36] Floß G., Saalfrank P. (2015). J. Phys. Chem. A.

