# Supplementary material for: Photoliquefaction and phase transition of m-bisazobenzenes give molecular solar thermal fuels with a high energy density
Source: RSC Adv. 2023 Aug 10;13(34):24031–7. doi: 10.1039/d3ra04595a (PMC10414017; doi:10.1039/d3ra04595a)
Supplement: RA-013-D3RA04595A-s001 [file RA-013-D3RA04595A-s001.pdf]

Electronic Supplementary Material (ESI) for RSC Advances.
This journal is © The Royal Society of Chemistry 2023

Photoliquefaction and phase transition of m-bisazobenzenes give molecular solar thermal fuels with a high energy density.

Masa-aki Morikawa,*[ab] Yuta Yamanaka,[a] Joseph Ka Ho Hui,[a] and Nobuo Kimizuka*[abc]

[a]*Department of Applied Chemistry, Graduate School of Engineering, Kyushu University,*

[b]*Center for Molecular Systems (CMS), Kyushu University,*

[c]*Research Center for Negative Emissions Technologies, Kyushu University (K-NETs), 744 Moto-oka, Nishi-ku, Fukuoka 819-0395*

Content

1. Synthesis of compounds 1-4……………………………………………………………S1

2. Supplementary figures………………………………………………………………..S4

3. Reference…………………………………………………………………………..……S26

1. Synthesis of compounds 1–4

Synthesis of 3-(4'-hydroxyphenylazo)acetanilide[1)]

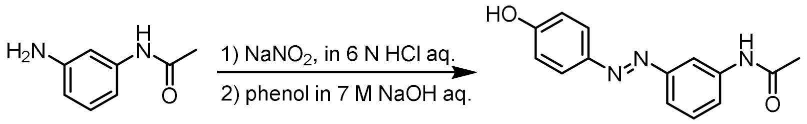

In a 100 mL beaker, 5.4 g of 3'-aminoacetanilide (36 mmol) was dissolved in 13 mL of water and 13 mL of 37% HCl. The mixture was stirred in an ice bath and 2.6 g of sodium nitrite (38 mmol) was added dropwise. The solution was left to stir for 30 min, while in a second 200 mL beaker, 3.3 g of phenol (35 mmol) and 7 g of NaOH (175 mmol) were dissolved in 25 mL of water. The contents of the first beaker were added dropwise to the second beaker and left to stir for 3 days. After neutralizing with 37% HCl, the resulting precipitate was filtered and washed with water. The crude product was purified by column chromatography of silica gel (ethyl acetate/hexane = 2/1, R_f = 0.3) to obtain an orange solid. Yield: 7.1 g, 77%. ^{1}H NMR (400 MHz, dmso-d) δ, ppm: 10.31 (s, 1H), 10.16 (s, 1H), 8.09 (t, J = 1.80 Hz, 1H), 7.79 (d, J = 8.80 Hz, 2H), 7.67 (dt, J = 7.60, 1.60 Hz, 1H), 7.52−7.45 (m, 2H), 6.95 (d, J = 9.20 Hz, 2H), 2.08 (s, 3H).

Synthesis of 3-(4'-hydroxyphenylazo)aniline[1)]

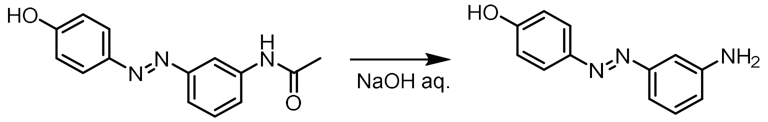

In a 500 mL round-bottom flask, 7.1 g of 3-(4'-hydroxyphenylazo)acetanilide (28 mmol) was suspended in 350 mL of 10% NaOH (875 mmol) aqueous solution and refluxed at 100 °C for 3 h. The mixture was then cooled to room temperature and the pH was adjusted to 6 with 37% HCl. The resulting precipitate was filtered, washed with water, and dried in vacuo to obtain an orange solid. The compound was sufficiently pure to be used in the subsequent step without further purification. Yield: 5.9 g, 100%. TLC (ethyl acetate/hexane = 2/1, R_f = 0.6).

Synthesis of 1,3-bis(4'-hydroxyphenylazo)benzene[1)]

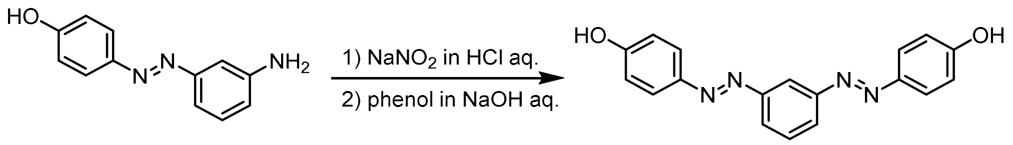

In a 200 mL beaker, 5.9 g of 3-(4'-hydroxyphenylazo)aniline (28 mmol) was placed, and 200 mL of water and 10 mL of 37% HCl were added. The mixture was stirred in an ice bath, and 1.9 g of sodium nitrite (28 mmol) in 10 mL of water was added dropwise. The solution was left to stir for 30 min to diazotize the aniline, while in a second 500 mL beaker, 2.6 g of phenol (28 mmol) and 5.6 g of NaOH (139 mmol) were dissolved in 50 mL of water. The contents of the first beaker were added dropwise to the second beaker and left to stir for one night. After neutralizing with 37% HCl, the precipitate was

filtered and washed with water. The crude product was purified by column chromatography with silica gel (ethyl acetate/hexane = 1/2, R_f = 0.2) to obtain a brown solid. Yield: 6.1 g, 69%. ^{1}H NMR (400 MHz, methanol-d4) δ, ppm: 8.26 (t, J = 2.00 Hz, 1H), 7.94 (dd, J = 7.80, 1.60 Hz, 2H), 7.88 (d, J = 8.80 Hz, 4H), 7.66 (t, J = 7.80 Hz, 1H), 6.94 (d, J = 8.80 Hz, 4H).

Synthesis of 1,3-bis((4-methoxyphenyl)diazenyl)benzene (1) and 4-((3-((4-methoxyphenyl)-diazenyl)phenyl)diazenyl)phenol (1a)

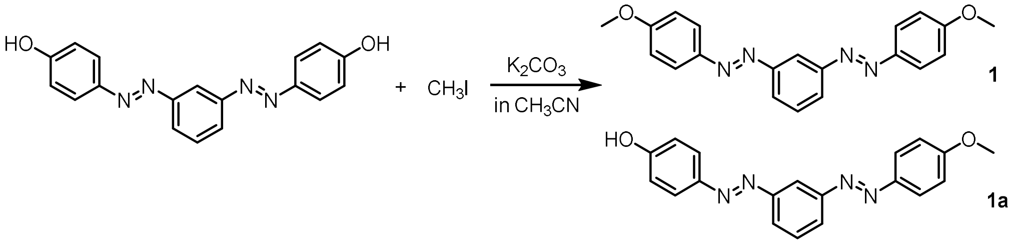

In a 500 mL three-neck flask, 3.0 g of 1,3-bis(4'-hydroxyphenylazo)benzene (9.4 mmol) and 2.6 g of K_2CO_3 (19 mmol) were placed. After replacing the air in the flask with nitrogen, 90 mL of dry acetonitrile and 1.3 g of iodomethane (9.4 mmol) were added, and the mixture was refluxed for 5 h. After removing the solvent, 100 mL of water was added, and the pH was adjusted to 6 with 37% HCl. The resulting precipitate was filtered and washed with water. The crude product was purified by column chromatography with silica gel (ethyl acetate/hexane = 1/2) to obtain **1** (R_f = 0.6) and **1a** (R_f = 0.4). Yield: 0.7 g, 22% (**1**), 1.6 g, 50% (**1a**). ^{1}H NMR (400 MHz, chloroform-d) δ, ppm (**1a**): 8.36 (t, J = 2.00 Hz, 1H), 7.98−7.96 (m, 4H), 7.93 (d, J = 8.80 Hz, 2H), 7.63 (t, J = 8.00 Hz, 1H), 7.04 (d, J = 9.20 Hz, 2H), 6.97 (d, J = 8.80 Hz, 2H), 5.31 (s, 1H), 3.91 (s, 3H), δ, ppm (**1**): 8.37 (t, J = 2.20 Hz, 1H), 7.99−7.96 (m, 6H), 7.63 (t, J = 7.80 Hz, 1H), 7.04 (d, J = 9.20 Hz, 4H), 3.91 (s, 6H). Elemental analysis for $C_{20}H_{18}N_4O_2$ (**1**): calculated (%) H 5.24, C 69.35, N 16.17; found (%) H 5.20, C 69.33, N 16.26.

Synthesis of 1-(4-ethoxyphenyl)-2-(3-((4-methoxyphenyl)diazenyl)phenyl)diazene (2)

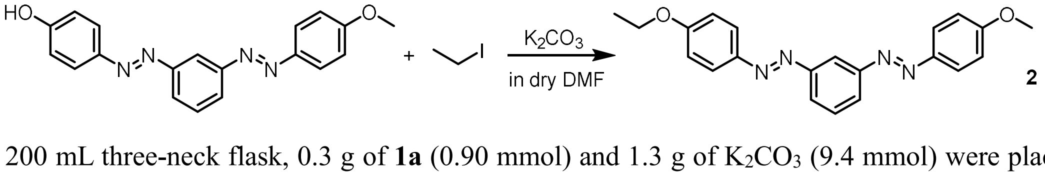

In a 200 mL three-neck flask, 0.3 g of **1a** (0.90 mmol) and 1.3 g of K_2CO_3 (9.4 mmol) were placed. After the air in the flask was replaced by nitrogen, 30 mL of dry DMF and 0.28 g of iodoethane (1.8 mmol) were added, and the mixture was stirred at 60 °C for 15 h. After removing DMF under reduced pressure, the product was extracted with DCM, washed with water several times, and dried over $MgSO_4$. The crude product was purified by silica gel column chromatography (DCM/hexane = 1/1, R_f

= 0.5) to obtain an orange solid. Yield: 0.27 g, 82%. ^{1}H NMR (400 MHz, chloroform-d) δ, ppm: 8.37 (t, J = 1.80 Hz, 1H), 7.99−7.94 (m, 6H), 7.63 (t, J = 8.00 Hz, 1H), 7.04 (d, J = 9.20 Hz, 2H), 7.02 (d, J = 8.40 Hz, 2H), 4.14 (q, J = 7.20 Hz, 2H), 3.91 (s, 3H), 1.47 (t, J = 7.2 Hz, 3H). Elemental analysis for $C_{21}H_{20}N_4O_2$: calculated (%) H 5.59, C 69.98, N 15.55; found (%) H 5.49, C 70.02, N 15.51.

Synthesis of 1-(4-ethoxyphenyl)-2-(3-((4-methoxyphenyl)diazenyl)phenyl)diazene (3)

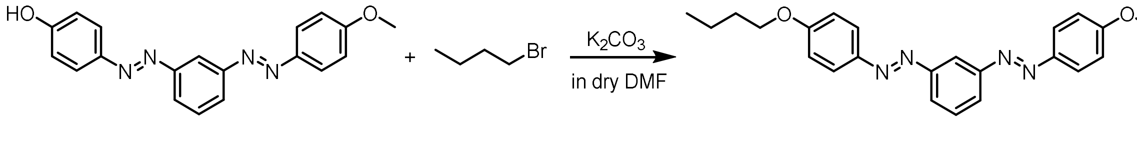

In a 200 mL three-neck flask, 0.7 g of **1a** (2.1 mmol) and 2.9 g of K_2CO_3 (21 mmol) were placed. After replacing the air in the flask with nitrogen, 30 mL of dry DMF and 0.38 g of 1-bromobutane (2.7 mmol) were added, and the mixture was stirred at 80 °C for 15 h. After removing DMF under reduced pressure, the product was extracted with DCM, washed with water several times, and dried over $MgSO_4$. The crude product was purified by silica gel column chromatography (DCM/hexane = 1/1, R_f = 0.5) to obtain an orange solid. Yield: 0.69 g, 84%. ^{1}H NMR (400 MHz, chloroform-d) δ, ppm: 8.37 (t, J = 1.80 Hz, 1H), 7.99−7.94 (m, 6H), 7.63 (t, J = 7.60 Hz, 1H), 7.04 (d, J = 9.20 Hz, 2H), 7.02 (d, J = 8.80 Hz, 2H), 4.07 (t, J = 6.60 Hz, 2H), 3.91 (s, 3H), 1.82 (quin, J = 7.00 Hz, 2H), 1.53 (sext, J = 7.47 Hz, 2H), 1.01 (t, J = 7.40 Hz, 3H). Elemental analysis for $C_{23}H_{24}N_4O_2$: calculated (%) H 6.23, C 71.11, N 14.42; found (%) H 6.27, C 71.16, N 14.41.

Synthesis of 1-(4-methoxyphenyl)-2-(3-((4-(neopentyloxy)phenyl)diazenyl)phenyl)diazene (4)

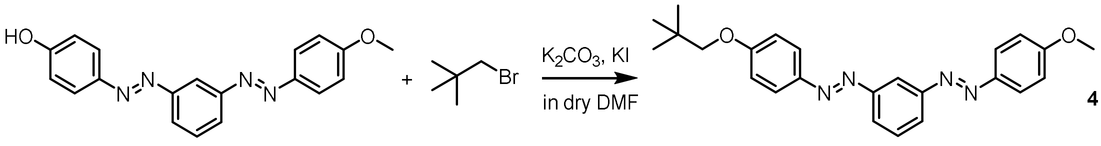

In a 200 mL three-neck flask, 0.74 g of **1a** (2.1 mmol) and 3.1 g of K_2CO_3 (22 mmol) were placed. After replacing the air in the flask with nitrogen, 30 mL of dry DMF, 0.18 g of KI (1.1 mmol), and 0.44 g of 1-bromo-2,2-dimethylpropane (2.9 mmol) were added, and the mixture was stirred at 120 °C for 20 h. After removing DMF under reduced pressure, the product was extracted with DCM, washed with water several times, and dried over $MgSO_4$. The crude product was purified by silica gel column chromatography (DCM/hexane = 1/2, R_f = 0.3) to obtain an orange solid. Yield: 0.75 g, 84%. ^{1}H NMR (400 MHz, chloroform-d) δ, ppm: 8.37 (t, J = 2.00 Hz, 1H), 7.99−7.94 (m, 6H), 7.63 (t, J = 8.00 Hz, 1H), 7.04 (d, J = 9.20 Hz, 2H), 7.03 (d, J = 9.20 Hz, 2H), 3.91 (s, 3H), 3.69 (s, 2H), 1.07 (s, 9H). Elemental analysis for $C_{24}H_{26}N_4O_2$: calculated (%) H 6.51, C 71.62, N 13.92; found (%) H 6.54, C 71.64, N 13.97.

2. Supplementary figures

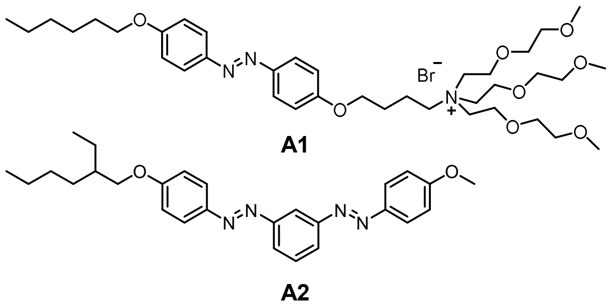

Scheme S1. Chemical structures of the derivative of ionic azobenzene (**A1**)[1] and liquid *m*-bisazobenzene (**A2**).[2]

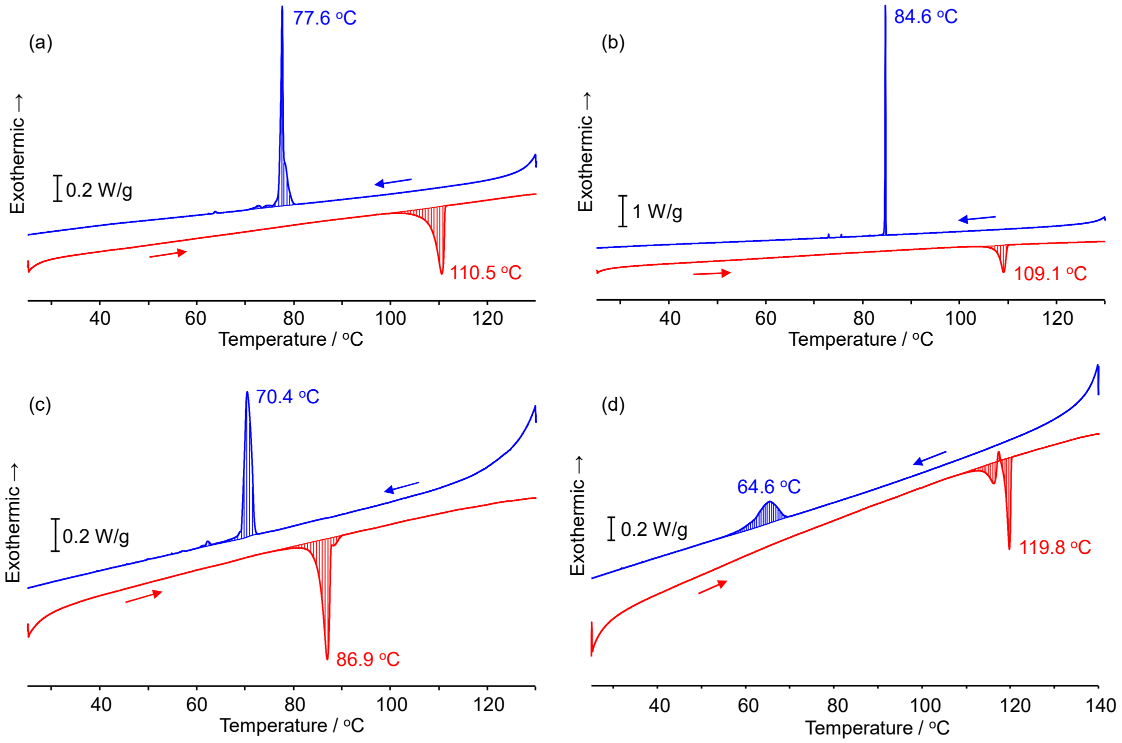

Figure S1. DSC thermograms of (a) (*E*,*E*)-**1**, (b) (*E*,*E*)-**2**, (c) (*E*,*E*)-**3** and (d) (*E*,*E*)-**4** in a heating and cooling cycle at 1 °C·min^{-1}.

Table S1. DSC data for bisazobenzenes (*E*,*E*)-**1**~**4**.

	Melting point (°C)	*ΔH* ($kJmol^{-1}$)	*ΔS* ($Jmol^{-1}K^{-1}$)
(*E*, *E*)-**1**	110.5	28.2	73.5
(*E*, *E*)-**2**	109.1	29.6	77.4
(*E*, *E*)-**3**	86.9	33.8	93.9
(*E*, *E*)-**4**	119.8	31.3	89.9

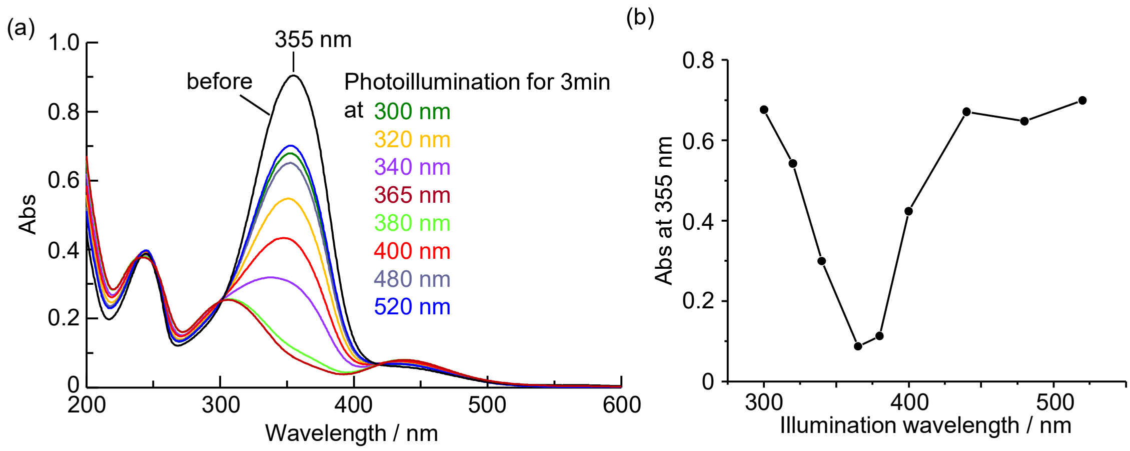

Figure S2. (a) UV-Vis absorption spectra of **1** in photostationary states under photoillumination with different wavelengths in acetonitrile (20 μM). (b) Dependence of the absorption intensity at 355 nm as a function of the photoillumination wavelength.

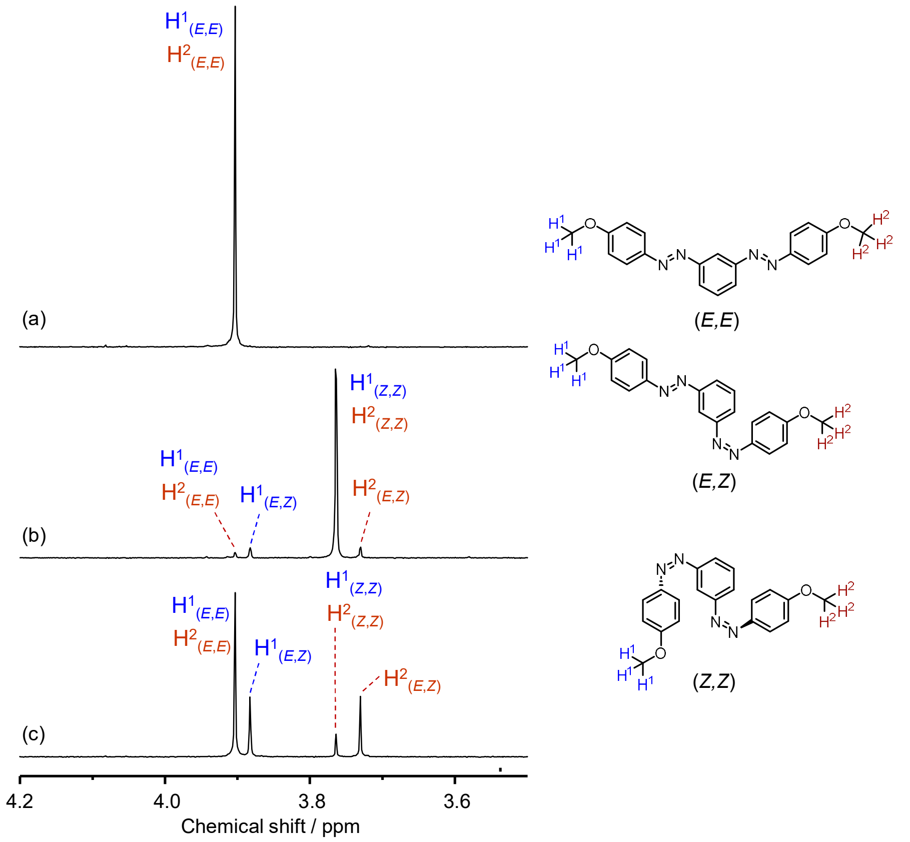

Figure S3. ^{1}H-NMR spectra of bisazobenzene **1** in CD_3CN at room temperature (concentration, 1 mM). (a) Before photoirradiation, after reaching the photostationary state excited at (b) 365 nm and (c) 520 nm, respectively.

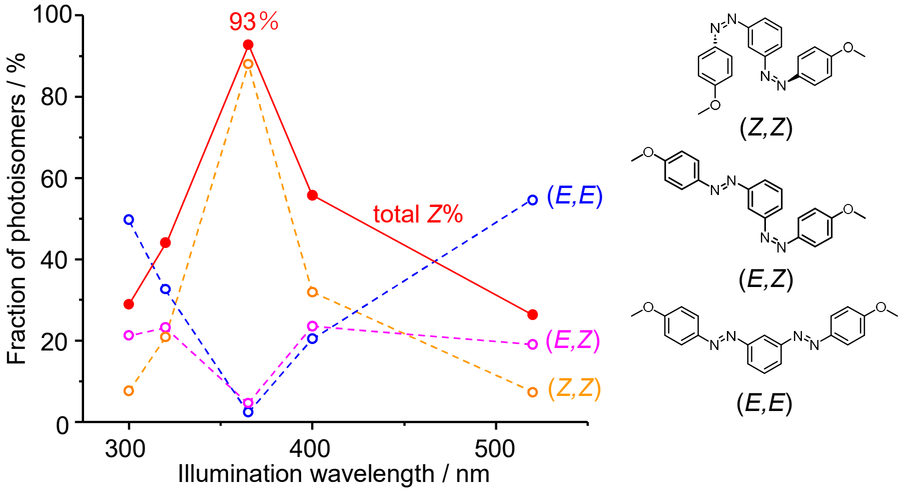

Figure S4. The fraction of the photoisomers of **1** as a function of the photoillumination wavelength determined by ^{1}H-NMR spectroscopy. The solid red circles indicate the total% of *Z* isomers. Photoillumination was carried out in CD_3CN, as shown in Figure S2.

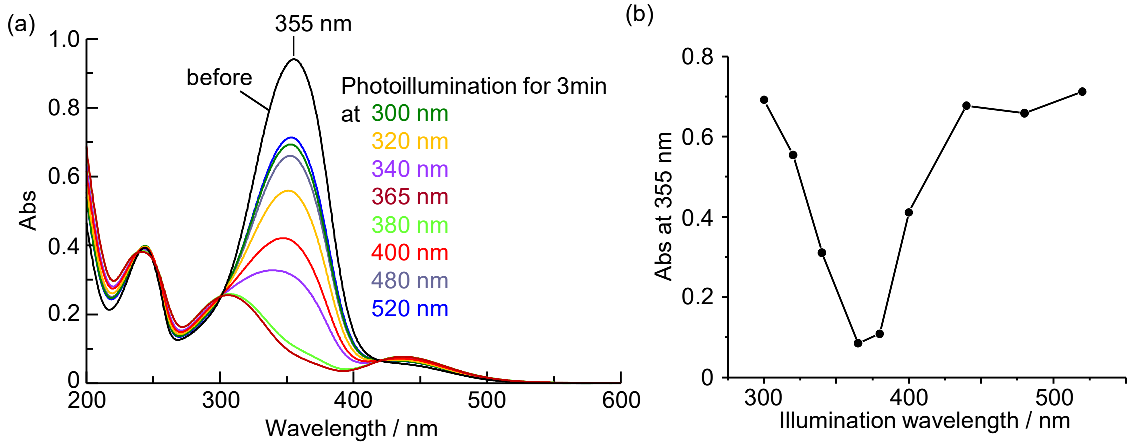

Figure S5. (a) UV-vis absorption spectra of **2** in photostationary states under photoillumination with different wavelengths in acetonitrile (20 μM). (b) Dependence of the absorption intensity at 355 nm as a function of the photoillumination wavelength.

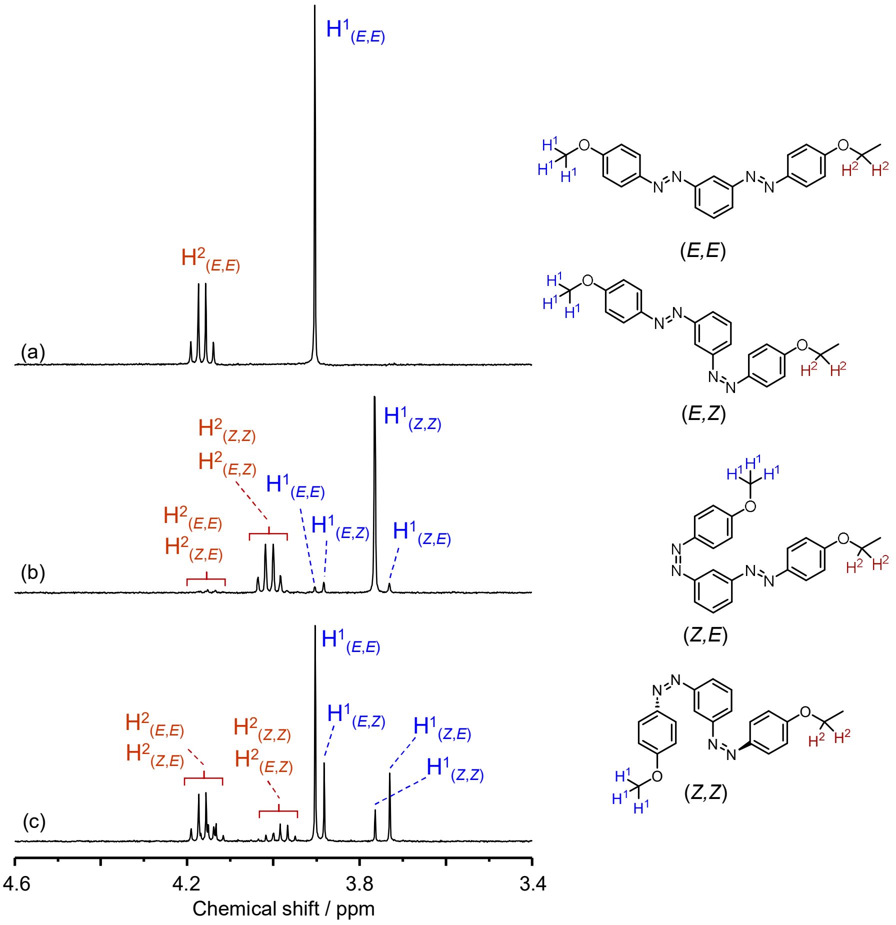

Figure S6. ^{1}H-NMR spectra of bisazobenzene **2** in CD_3CN at room temperature (concentration, 1 mM). (a) Before photoirradiation, after reaching the photostationary state excited at (b) 365 nm and (c) 520 nm, respectively.

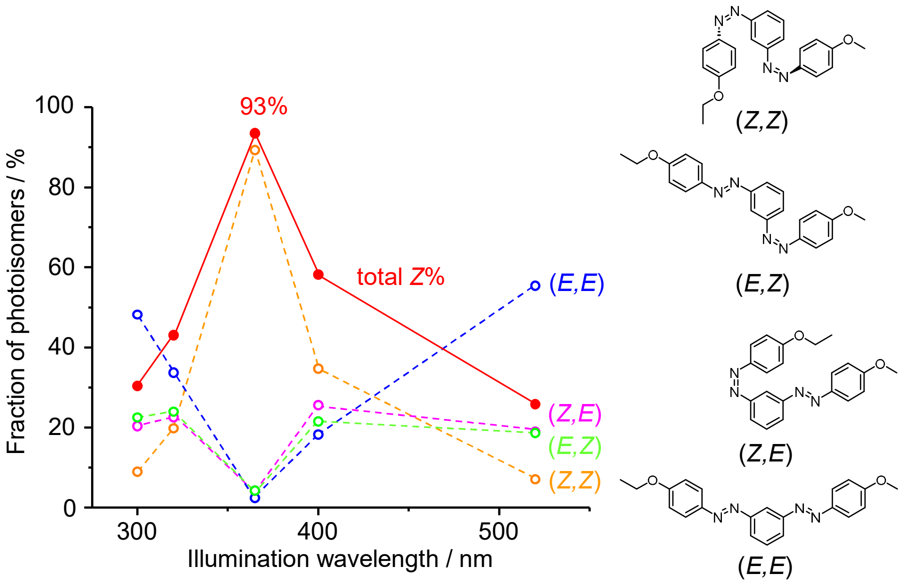

Figure S7. The photoisomer fraction of **2** as a function of the photoillumination wavelength determined by ^{1}H-NMR spectroscopy. The solid red circles indicate the total% of *Z* isomers. Photoillumination was performed in CD_3CN, as shown in Figure S5.

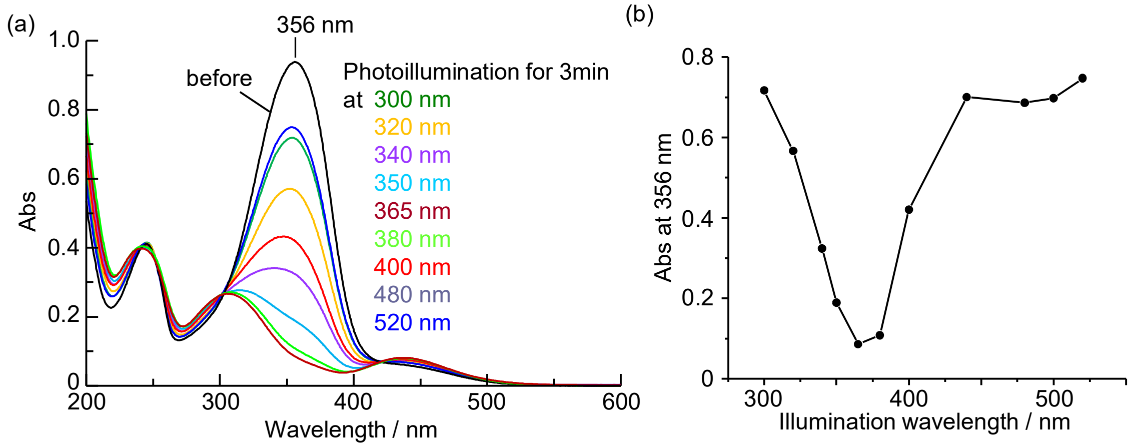

Figure S8. (a) UV-Vis absorption spectra of **3** in photostationary states under photoillumination with different wavelengths in acetonitrile (20 μM). (b) Dependence of the absorption intensity at 356 nm as a function of the photoillumination wavelength.

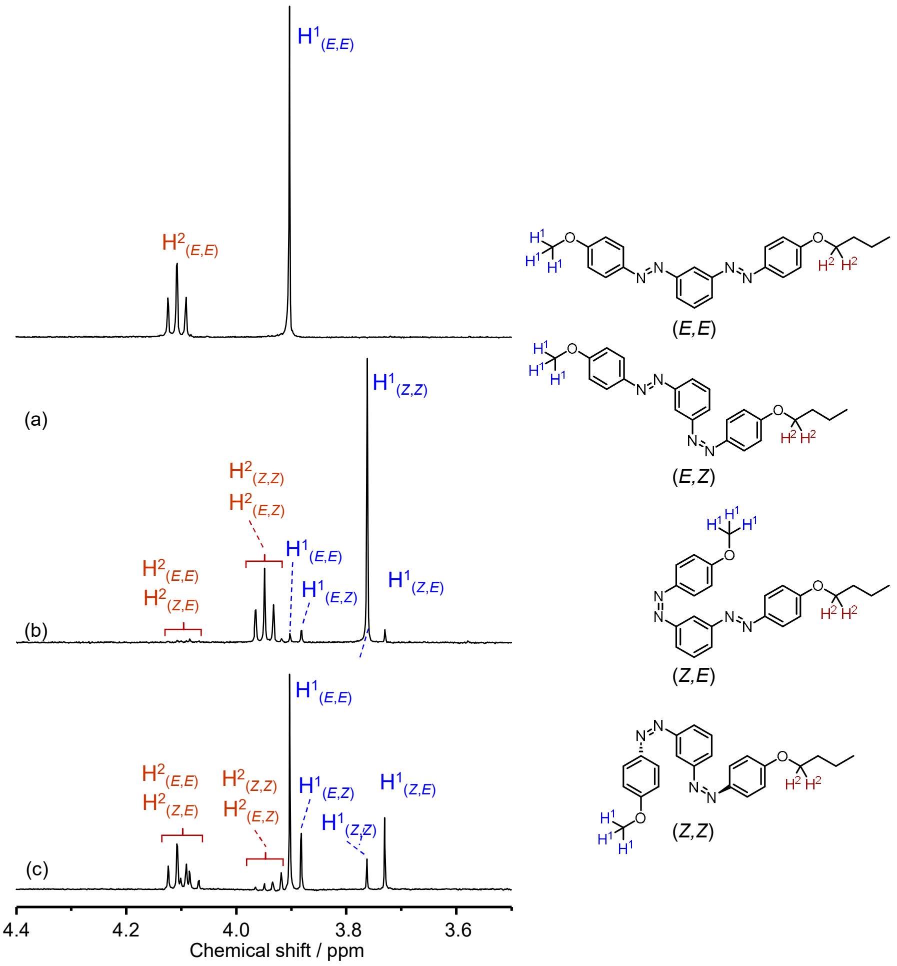

Figure S9. ^{1}H-NMR spectra of bisazobenzene **3** in CD_3CN at room temperature (concentration, 1 mM). (a) before photoirradiation, after reaching the photostationary state excited at (b) 365 nm and (c) 520 nm, respectively.

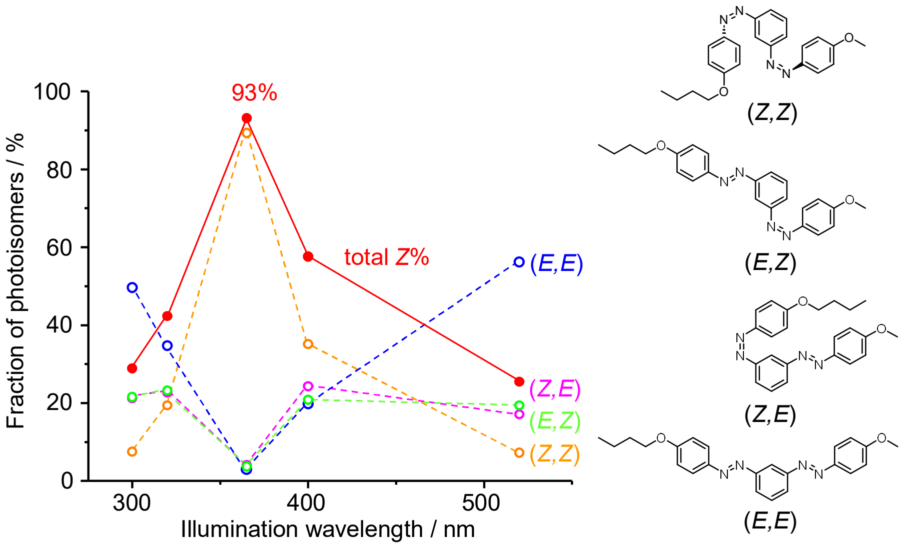

Figure S10. The fraction of the photoisomers of **3** as a function of the photoillumination wavelength determined by ^{1}H-NMR spectroscopy. The solid red circles indicate the total% of *Z* isomers. Photoillumination was carried out in CD_3CN, as shown in Figure S8.

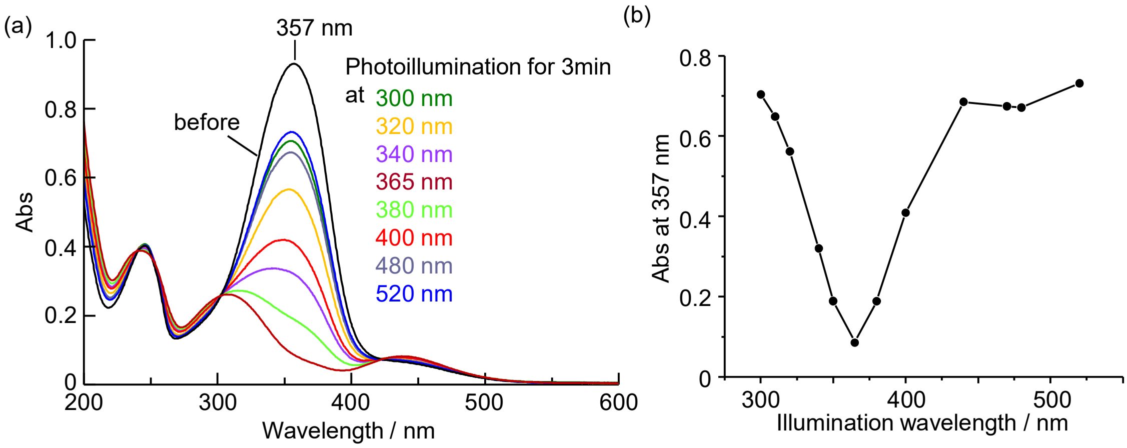

Figure S11. (a) UV-vis absorption spectra of **4** in photostationary states under photoillumination with different wavelengths in acetonitrile (20 μM). (b) Dependence of the absorption intensity at 357 nm as a function of the photoillumination wavelength.

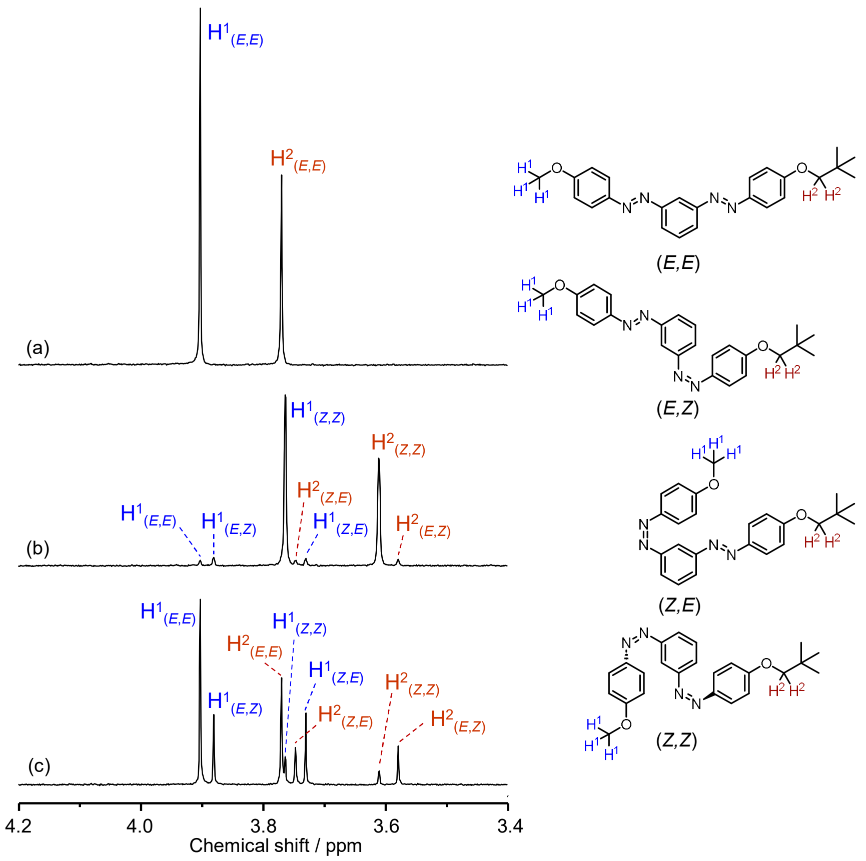

Figure S12. ^{1}H-NMR spectra of bisazobenzene **4** in CD_3CN at room temperature (concentration, 1 mM). (a) Before photoirradiation, after reaching the photostationary state excited at (b) 365 nm and (c) 520 nm, respectively.

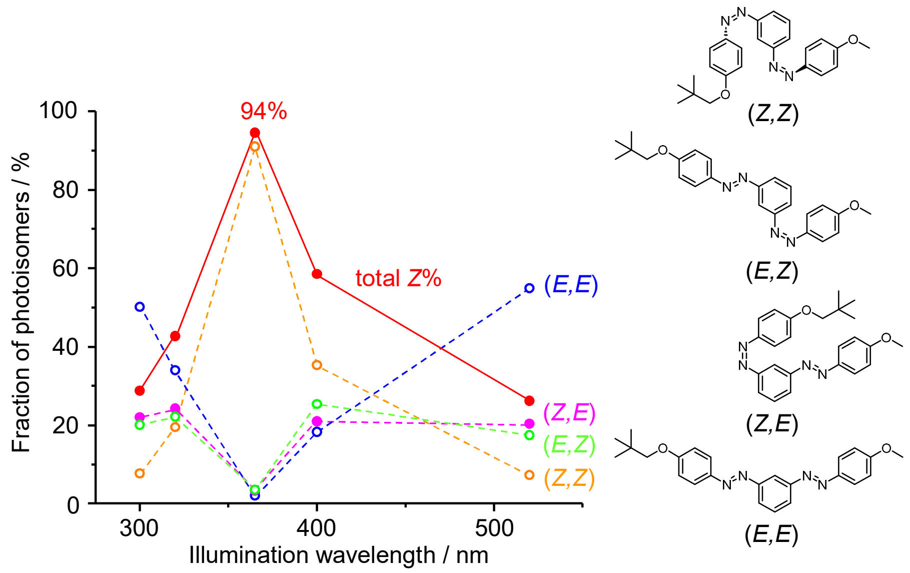

Figure S13. The fraction of photoisomers of **4** as a function of the photoillumination wavelength determined by ^{1}H-NMR spectroscopy. The solid red circles indicate the total% of *Z* isomers. Photoillumination was performed in CD_3CN, as shown in Figure S11.

Table S2. Absorption and photoisomerization parameters in solution.

	S_0-S_2[a] λ_{max} / nm (ε / M^{-1} cm^{-1})	S_0-S_1[a] λ_{max} / nm (ε / M^{-1} cm^{-1})	*Z*% PSS[a, b]	*E*% PSS[a, c]
(*E*, *E*)-**1**	355 (45,200)	436 (3,000)	93	74
(*E*, *E*)-**2**	355 (47,000)	436 (2,800)	93	74
(*E*, *E*)-**3**	356 (46,900)	436 (3,000)	93	75
(*E*, *E*)-**4**	357 (46,500)	436 (3,300)	94	74

[a] in acetonitrile, at 25 ˚C. [b] Percentage of (*Z*)-azo units at the PSS. Illumination wavelength of maximum conversion was 365 nm. [c] Percentage of (*E*)-azo units at the PSS. Illumination wavelength of maximum conversion was 520 nm.

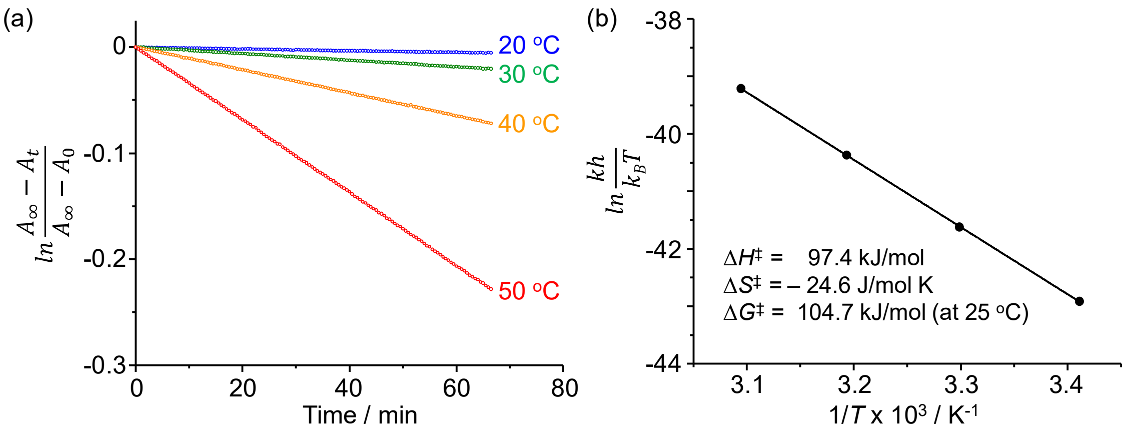

Figure S14. (a) First-order plots and (b) Eyring plots for the *Z*-to-*E* thermal isomerization of bisazobenzene **1** in acetonitrile solution (concentration; 20 μM).

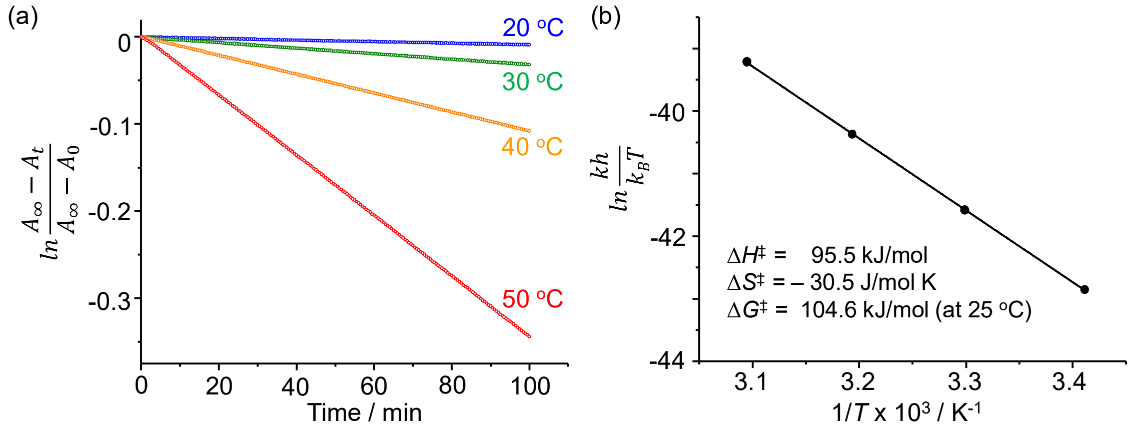

Figure S15. (a) First-order plots and (b) Eyring plots for the *Z*-to-*E* thermal isomerization of bisazobenzene **2** in acetonitrile solution (concentration; 20 μM).

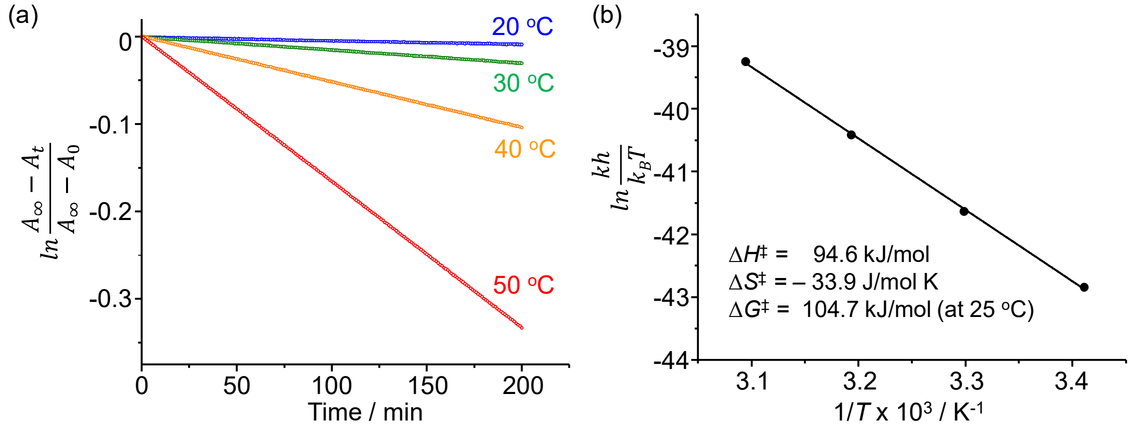

Figure S16. (a) First-order plots and (b) Eyring plots for the *Z*-to-*E* thermal isomerization of bisazobenzene **3** in acetonitrile solution (concentration; 20 μM).

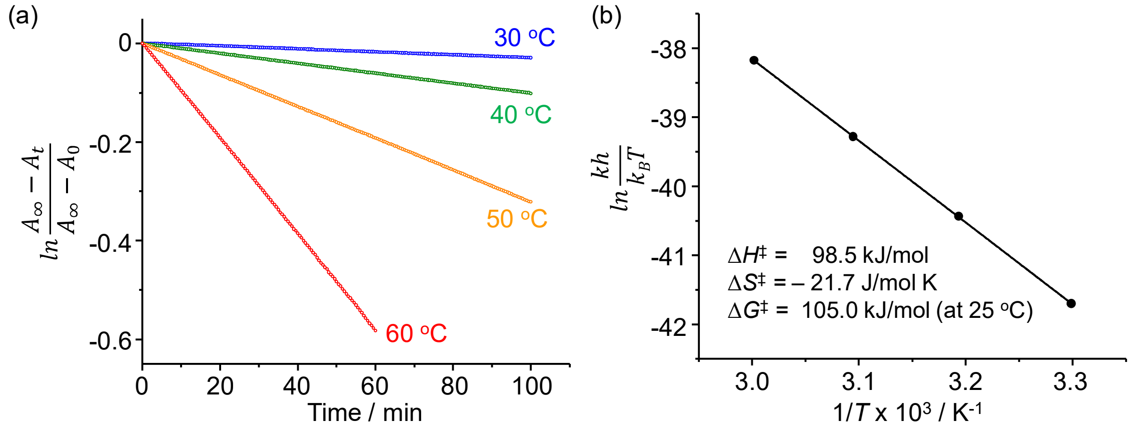

Figure S17. (a) First-order plots and (b) Eyring plots for the *Z*-to-*E* thermal isomerization of bisazobenzene **4** in acetonitrile solution (concentration; 20 μM).

Table S3. The activation parameters of the *Z*-to-*E* thermal isomerization for bisazobenzenes **1**–**4**.

	$\Delta G^{\ddagger}$ at 25 °C ($kJmol^{-1}$)	$t_{1/2}$ (day)	$\Delta H^{\ddagger}$ ($kJmol^{-1}$)	$\Delta S^{\ddagger}$ ($Jmol^{-1}K^{-1}$)
**1**	104.7 ± 0.4	2.9	97.4 ± 0.2	-24.6 ± 0.7
**2**	104.6 ± 0.6	2.8	95.5 ± 0.3	-30.5 ± 1.0
**3**	104.7 ± 1.4	2.9	94.6 ± 0.7	-33.9 ± 2.4
**4**	105.0 ± 0.3	3.2	98.5 ± 0.2	-21.7 ± 0.5

The activation free energies ($\Delta G^{\ddagger}$) of compounds **1-4** at 25 °C were determined to be around 105 $kJ \cdot mol^{-1}$ (Table S3), which are similar to those of pristine azobenzenes.[3]

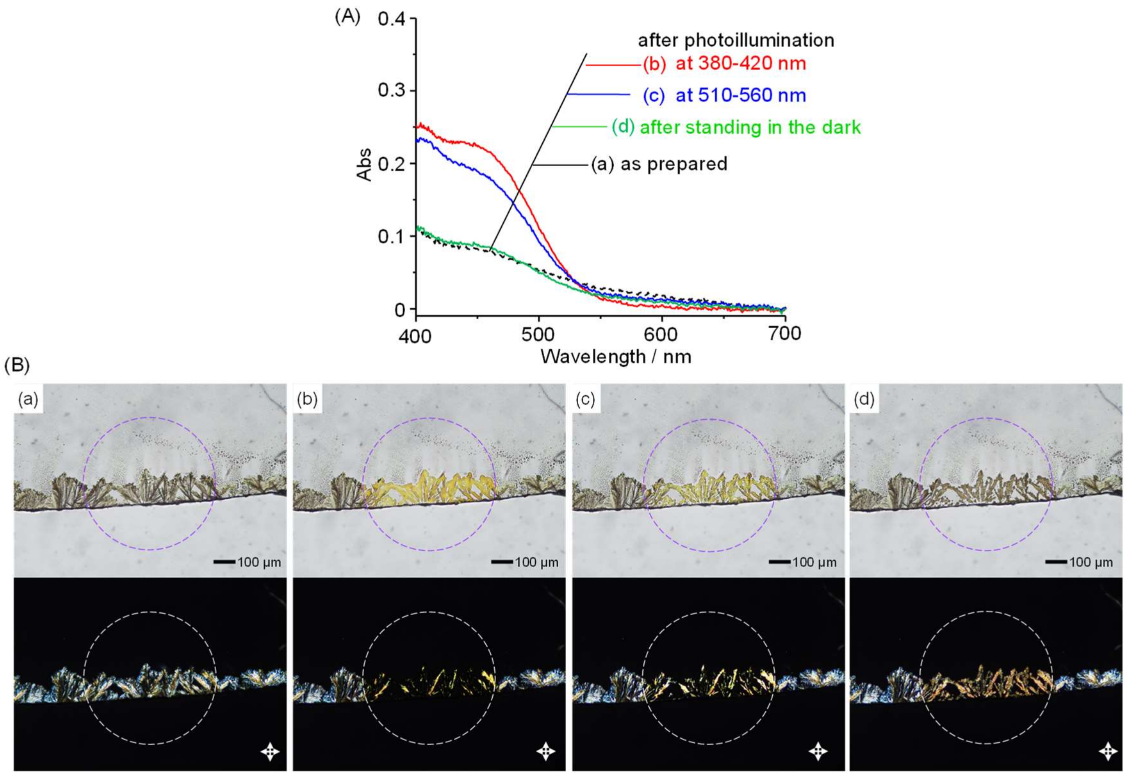

Figure S18. (A) Visible absorption spectra and (B) optical microscopy images (top; bright field, bottom; under crossed polarizers) of drop cast film (*E*,*E*)-**2** on a glass substrate. (a) as prepared, (b, c) after photoillumination with mercury lamp for 5 sec with bandpass filters: (b) 380-420 nm, (c) 510-560 nm, and (d) after standing for 5 min in the dark.

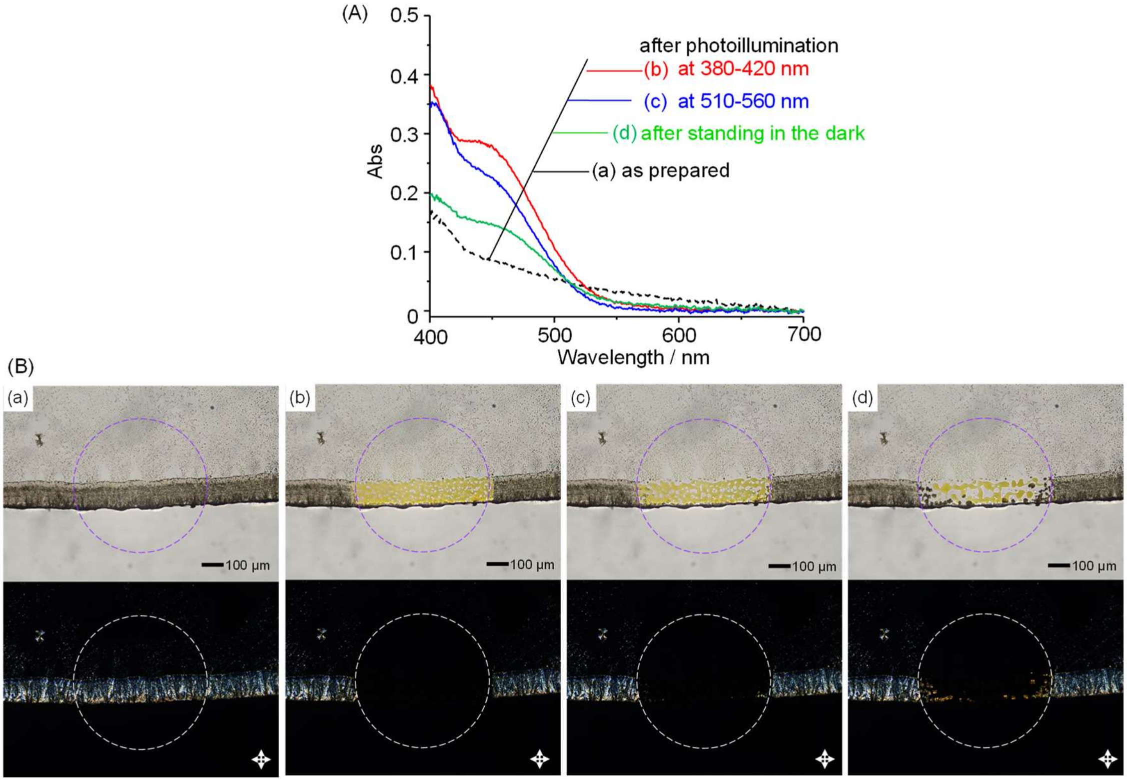

Figure S19. (A) Visible absorption spectra and (B) optical microscopy images (top; bright field, bottom; under crossed polarizers) of drop-cast film (*E*,*E*)-**3** on a glass substrate. (a) as prepared, (b, c) after photoillumination with mercury lamp for 5 sec with bandpass filters: (b) 380-420 nm, (c) 510-560 nm, and (d) after standing for 5 min in the dark.

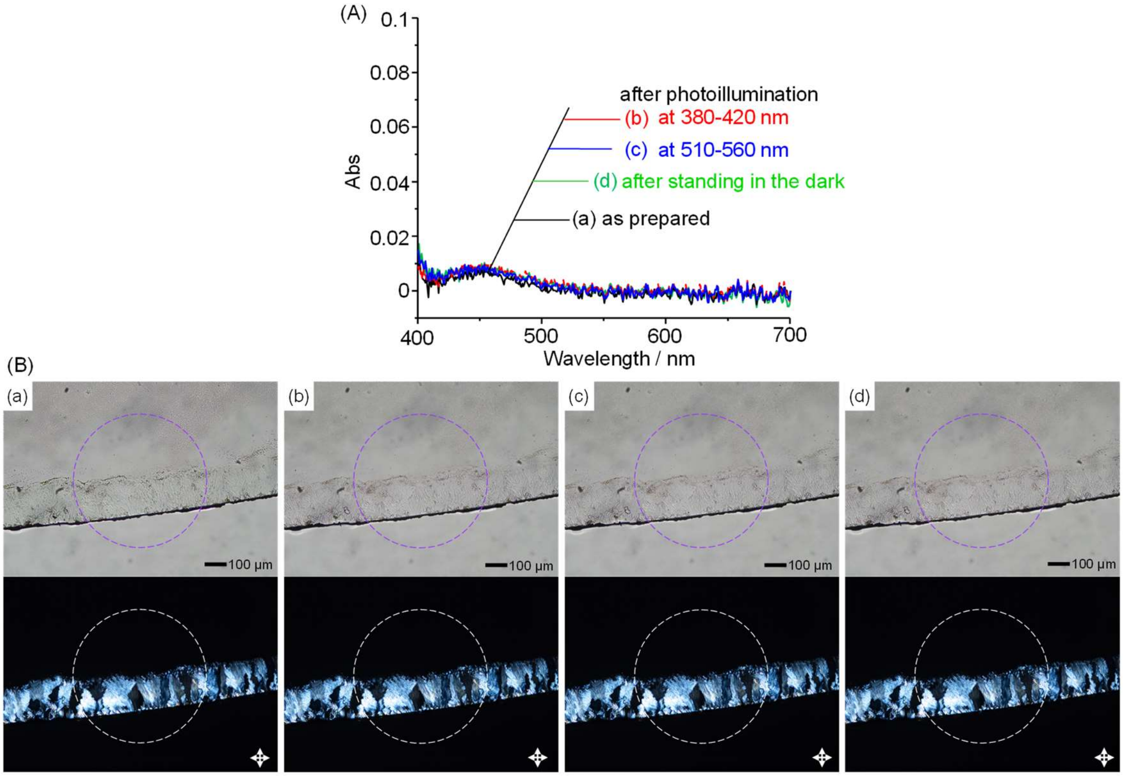

Figure S20. (A) Visible absorption spectra and (B) optical microscopy images (top; bright field, bottom; under crossed polarizers) of drop cast film (*E*,*E*)-**4** on a glass substrate. (a) as prepared, (b, c) after photoillumination with mercury lamp for 5 sec with bandpass filters: (b) 380-420 nm, (c) 510-560 nm, and (d) after standing for 5 min in the dark.

(a)

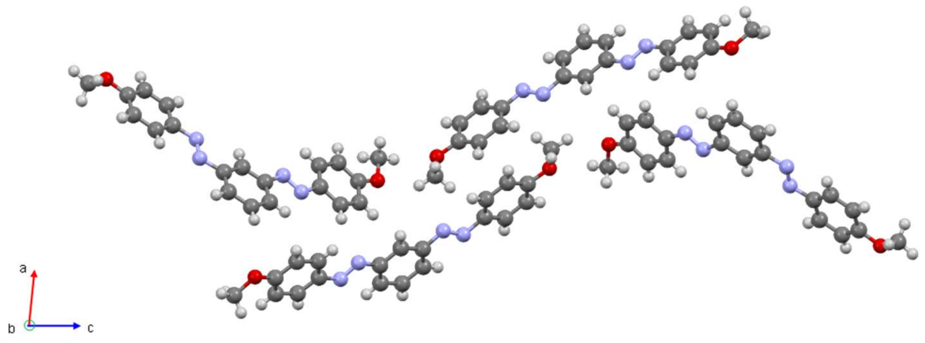

(b)

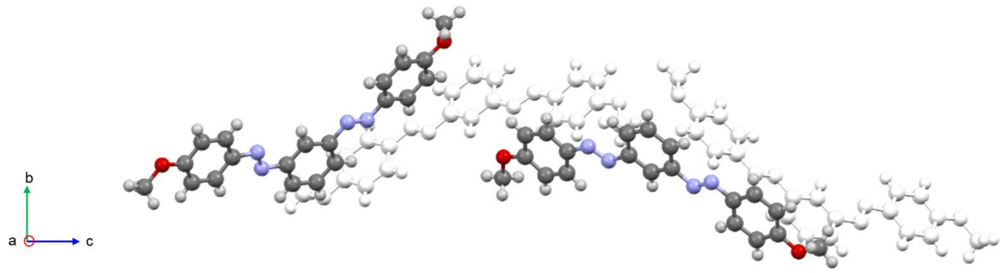

Figure S21. The unit cell structure of bisazobenzene (*E*,*E*)-**1** determined by single-crystal XRD analysis.

(a) ac plane, (b) bc plane. A single crystal suitable for measurement was obtained from EtOH.

Formula	C_{20} H_{18} N_4 O_2
Space group	$P2_1$
Cell length	a(Å) 9.3875(2)
	b(Å) 5.35880(10)
	c(Å) 34.5503(7)
Cell angles	α(°) 90
	β(°) 92.723(2)
	γ(°) 90
Cell volume	1736.12
Density	1.325 g cm^{-3}
Z	4
R-Factor(%)	3.6

(a)

(b)

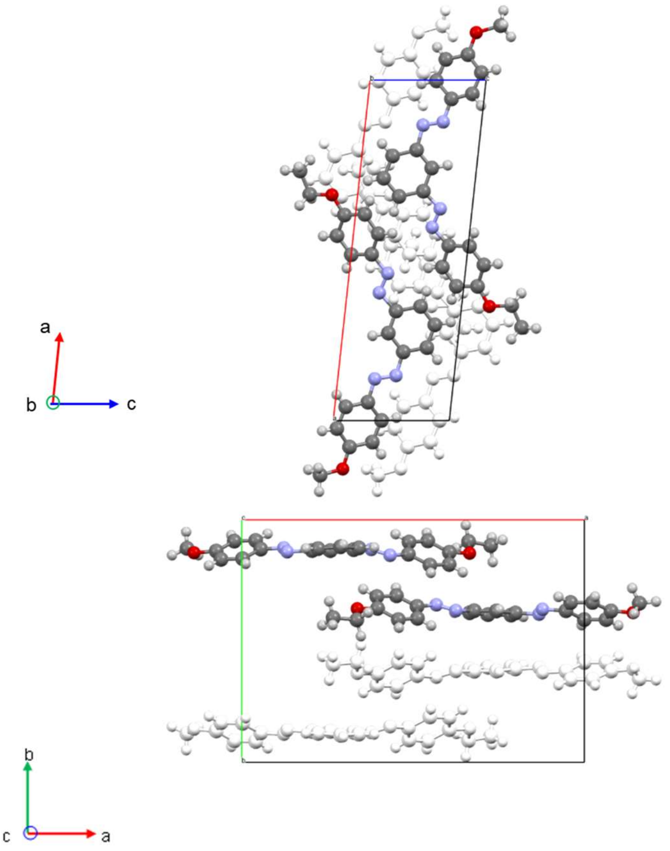

Figure S22. The solid-state structure of bisazobenzene (*E*,*E*)-**2** determined by single-crystal XRD analysis. (a) ac plane, (b) ab plane, A single crystal suitable for measurement was obtained from EtOH.

Formula	$C_{21} H_{20} N_4 O_2$
Space group	Pc
Cell length	a(Å) 19.7321(12)
	b(Å) 13.8945(5)
	c(Å) 6.6817(3)
Cell angles	α(°) 90
	β(°) 96.168(5)
	γ(°) 90
Cell volume	1821.3
Density	1.314 g cm^{-3}
Z	4
R-Factor(%)	11.22

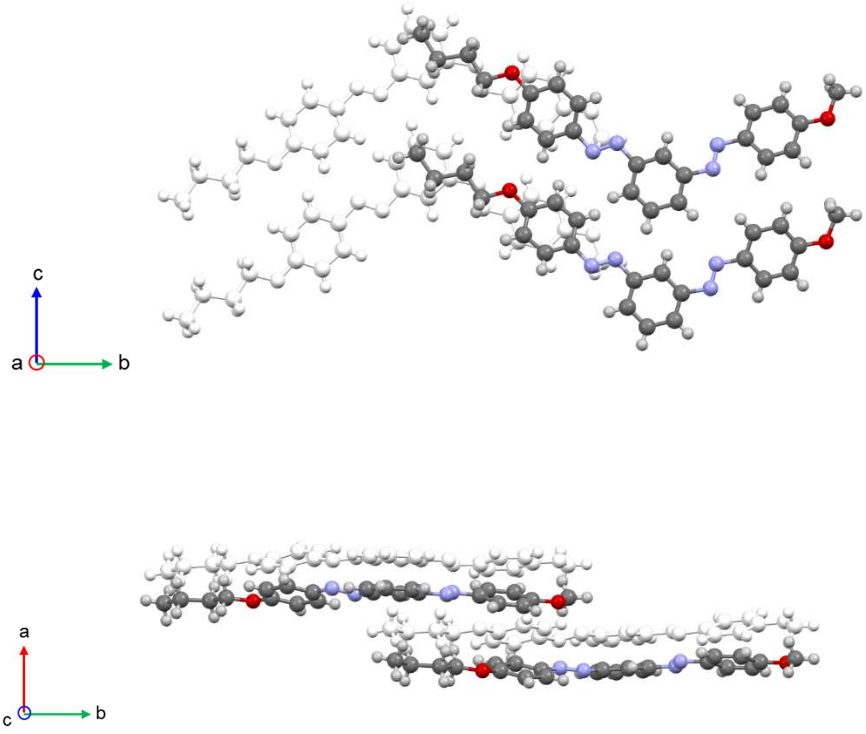

Figure S23. Solid-state structure of bisazobenzene (*E*,*E*)-**3** determined by single-crystal XRD analysis. (a) ac plane, (b) ab plane, A single crystal suitable for measurement was obtained from EtOH.

Formula	$C_{23}\ H_{24}\ N_4\ O_2$	
Space group	$P2_1$	
Cell length	a(Å)	7.3869(3)
	b(Å)	23.1029(7)
	c(Å)	11.7353(5)
Cell angles	α(°)	90
	β(°)	93.452(4)
	γ(°)	90
Cell volume	1999.1	
Density	1.291 g cm^{-3}	
Z	4	
R-Factor(%)	16.14	

Good single crystals were not obtained for compounds (*E*,*E*)-**2** and (*E*,*E*)-**3**; therefore, the R values did not reach satisfactory small values. We show these data in Fig. S22 and S23 as references.

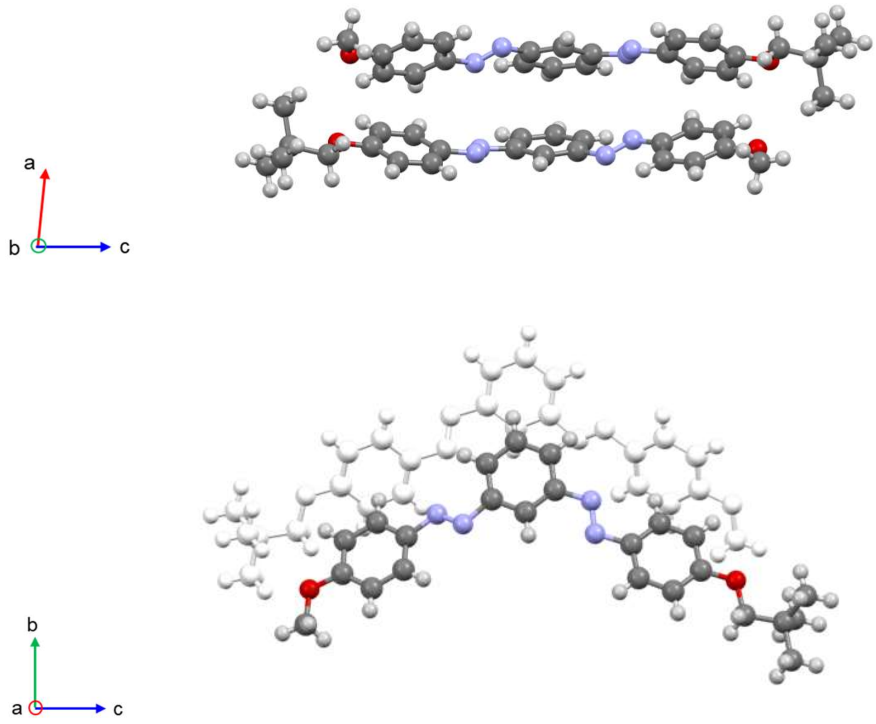

Figure S24. The solid-state structure of bisazobenzene (*E*,*E*)-**4** determined by single-crystal XRD analysis. (a) ac plane, (b) bc plane. A single crystal suitable for measurement was obtained from EtOH.

Formula	C_{24} H_{26} N_4 O_2
Space group	$P2_1$
Cell length	a(Å) 7.10900(10)
	b(Å) 6.35350(10)
	c(Å) 23.4700(6)
Cell angles	α(°) 90
	β(°) 95.487(2)
	γ(°) 90
Cell volume	1055.21
Density	1.267 g cm^{-3}
Z	2
R-Factor(%)	4.12

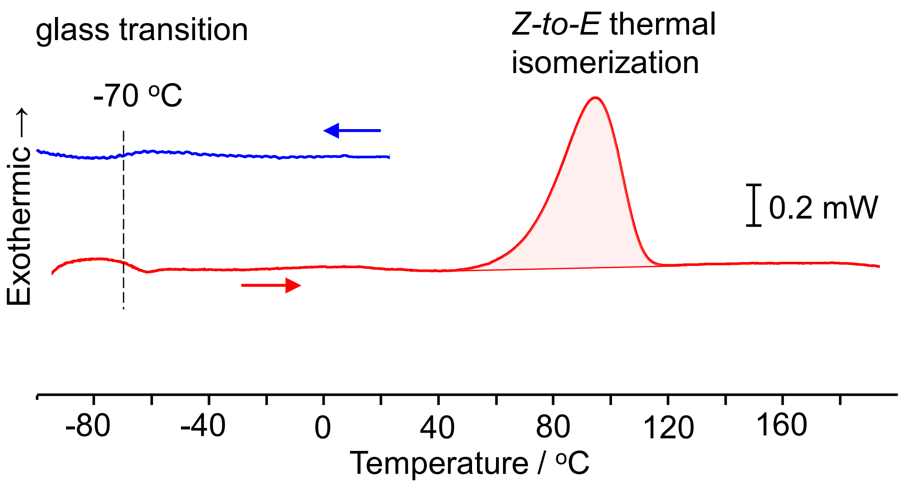

Figure S25. DSC thermograms of **1** after photoisomerization by UV light irradiation. The total *Z*% (87.5%) was determined by ^{1}H-NMR spectroscopy. The blue and red lines correspond to the cooling and subsequent heating processes: scan rate, 5 °C·min^{-1}. The *Z*-rich compound **1** was cooled down to about -95°C (blue line), and then the heating scan (red line) was recorded. Only grass transition peaks were obtained around -70 ˚C in both of the cooling and the subsequent heating process. The absence of endothermic peaks associated with crystallization indicates that the *Z*-**1** is essentially in the liquid state.

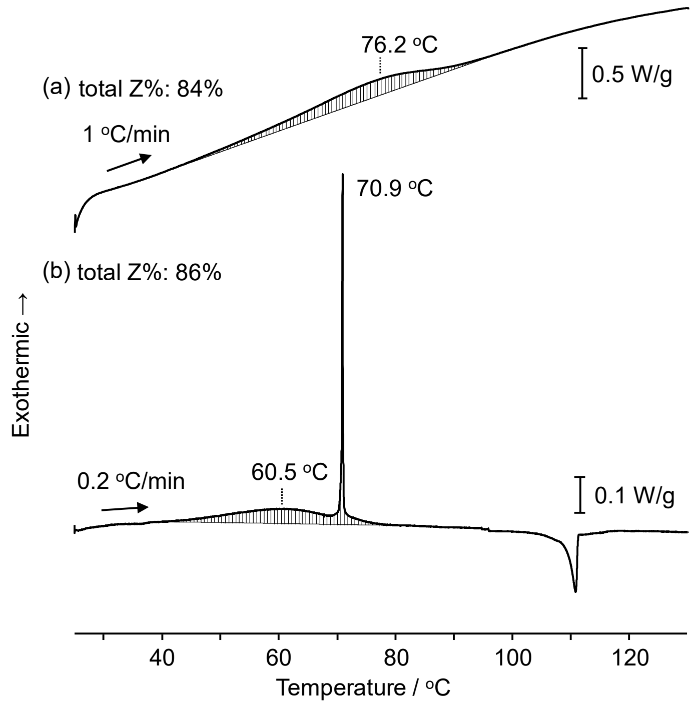

Figure S26. DSC thermograms of **1** after UV light irradiation at a scan rate of (a) 1 °C·min^{-1} and (b) 0.2°C·min^{-1}, respectively. Total *Z*% was determined by ^{1}H-NMR spectroscopy.

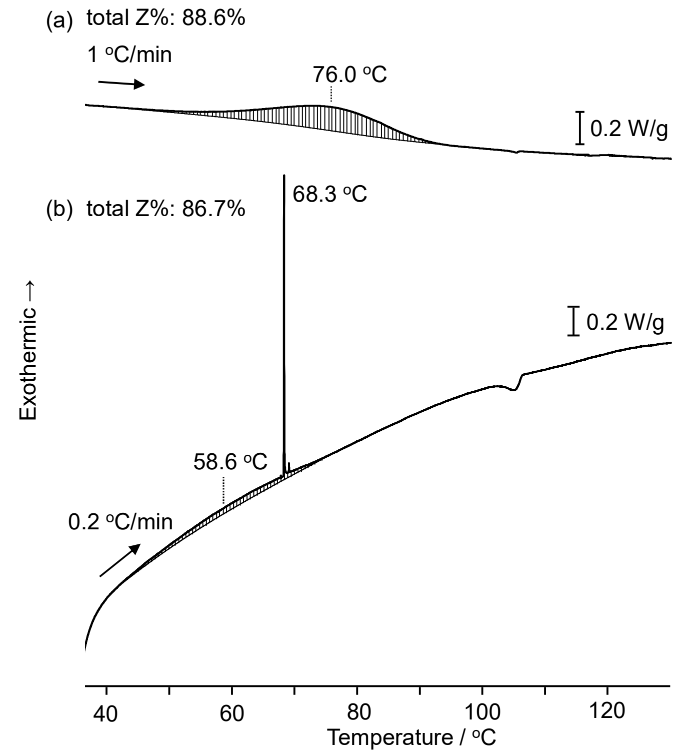

Figure S27. DSC thermograms of **2** after UV light irradiation at a scan rate of (a) 1 °C·min^{-1} and (b) 0.2 °C·min^{-1}, respectively. Total *Z*% was determined by ^{1}H-NMR spectroscopy.

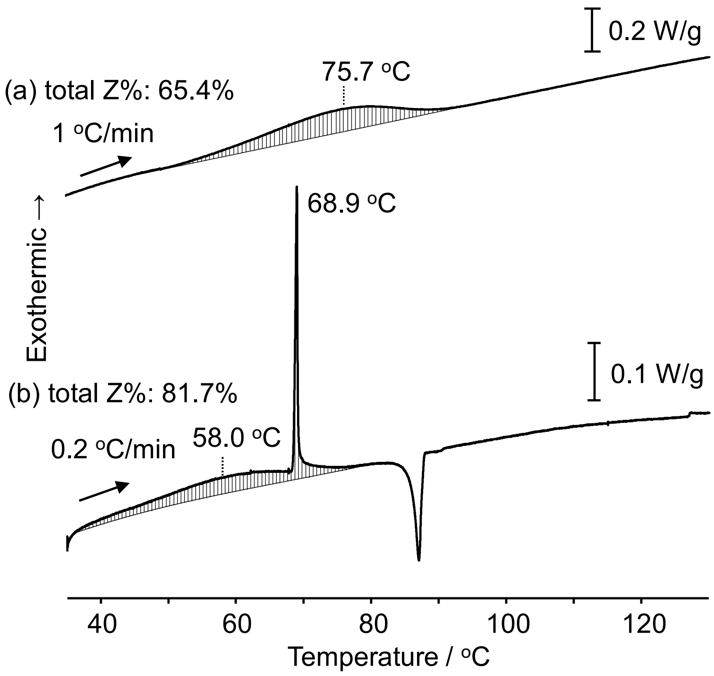

Figure S28. DSC thermograms of **3** after UV light irradiation at a scan rate of (a) 1 °C/min and (b) 0.2°C/min, respectively. Total *Z*% was determined by ^{1}H-NMR spectroscopy.

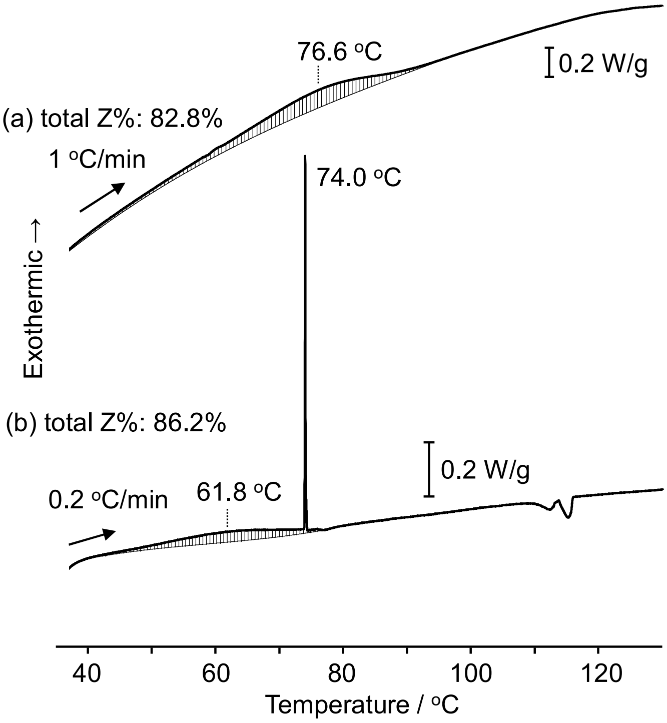

Figure S29. DSC thermograms of **4** after UV light irradiation at a scan rate of (a) 1 °C/min and (b) 0.2°C/min, respectively. Total *Z*% was determined by ^{1}H-NMR spectroscopy.

Table S4. The isomerization enthalpy (ΔH_{isom}) and the cystallization enthalpy (ΔH_c) of bisazobenzenes **1**–**4** and two reported compounds (**A1** and **A2**).

	ΔH_{isom}		ΔH_c		$\Delta H_{isom} + \Delta H_c$	
	(kJ/mol)	(J/g)	(kJ/mol)	(J/g)	(kJ/mol)	(J/g)
**1**	102.7	296.5	33.1	95.5	135.8	392.0
**2**	112.8	313.1	20.5	56.8	133.3	369.9
**3**	101.1	260.3	30.9	79.6	132.0	339.9
**4**	110.9	275.7	21.3	52.8	132.2	328.5
**A1**	51.8	68.4	57.3	75.7	109.1	144.1
**A2**	102.2	230.0	0	0	102.2	230.0

ΔH_{isom} and ΔH_c were determined from the exothermic peaks in the first heating scan of the DSC thermograms. These values were calculated as 100% for the *Z*-isomer contents.

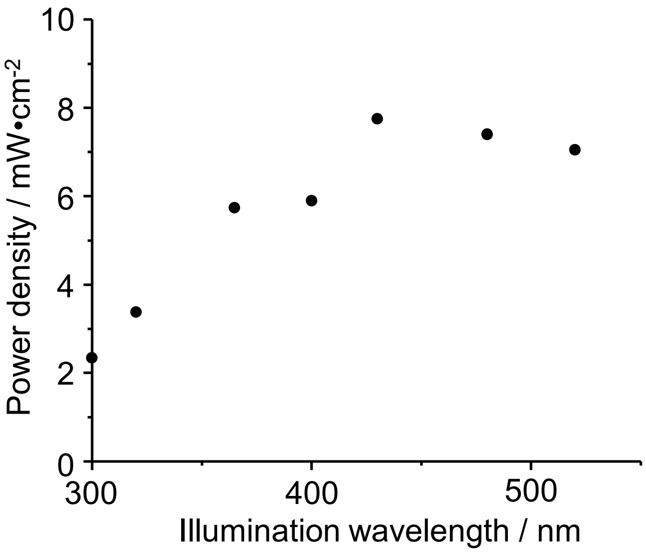

Figure S30. The power density of the xenon ramp (MAX-350, Asahi Spectra) as a function of the illumination wavelength. Bandpass filters (FWHM: 10 nm) were employed to irradiate light of various wavelengths.

4. References

1) K. Ishiba, M-a. Morikawa, C. Chikara, T. Yamada, K. Iwase, M. Kawakita, N. Kimizuka, *Angew. Chem. Int. Ed*. **2015,** *54*, 1532.

2) M-a. Morikawa, Y. Yamanaka, N. Kimizuka, *Chem. Lett*. **2022**, *51*, 402.

3) E. V. Brown, G. R. Granneman, *J. Am. Chem. Soc*. **1975**, *97*, 621.
